# The Genetic Architecture of Natural Variation in Recombination Rate in *Drosophila melanogaster*

**DOI:** 10.1371/journal.pgen.1005951

**Published:** 2016-04-01

**Authors:** Chad M. Hunter, Wen Huang, Trudy F. C. Mackay, Nadia D. Singh

**Affiliations:** 1 Program in Genetics, Department of Biological Sciences, North Carolina State University, Raleigh, North Carolina, United States of America; 2 W. M. Keck Center for Behavioral Biology, North Carolina State University, Raleigh, North Carolina, United States of America; 3 Initiative in Biological Complexity, North Carolina State University, Raleigh, North Carolina, United States of America; 4 Bioinformatics Research Center, North Carolina State University, Raleigh, North Carolina, United States of America; The University of North Carolina at Chapel Hill, UNITED STATES

## Abstract

Meiotic recombination ensures proper chromosome segregation in many sexually reproducing organisms. Despite this crucial function, rates of recombination are highly variable within and between taxa, and the genetic basis of this variation remains poorly understood. Here, we exploit natural variation in the inbred, sequenced lines of the *Drosophila melanogaster* Genetic Reference Panel (DGRP) to map genetic variants affecting recombination rate. We used a two-step crossing scheme and visible markers to measure rates of recombination in a 33 cM interval on the *X* chromosome and in a 20.4 cM interval on chromosome *3R* for 205 DGRP lines. Though we cannot exclude that some biases exist due to viability effects associated with the visible markers used in this study, we find ~2-fold variation in recombination rate among lines. Interestingly, we further find that recombination rates are uncorrelated between the two chromosomal intervals. We performed a genome-wide association study to identify genetic variants associated with recombination rate in each of the two intervals surveyed. We refined our list of candidate variants and genes associated with recombination rate variation and selected twenty genes for functional assessment. We present strong evidence that five genes are likely to contribute to natural variation in recombination rate in *D*. *melanogaster*; these genes lie outside the canonical meiotic recombination pathway. We also find a weak effect of Wolbachia infection on recombination rate and we confirm the interchromosomal effect. Our results highlight the magnitude of population variation in recombination rate present in *D*. *melanogaster* and implicate new genetic factors mediating natural variation in this quantitative trait.

## Author Summary

During meiosis, homologous chromosomes exchange genetic material through recombination. In most sexually reproducing species, recombination is necessary for chromosomes to properly segregate. Recombination defects can generate gametes with an incorrect number of chromosomes, which is devastating for organismal fitness. Despite the central role of recombination for chromosome segregation, recombination is highly variable process both within and between species. Though it is clear that this variation is due at least in part to genetics, the specific genes contributing to variation in recombination within and between species remain largely unknown. This is particularly true in the model organism, *Drosophila melanogaster*. Here, we use the *D*. *melanogaster* Genetic Reference Panel to determine the scale of population-level variation in recombination rate and to identify genes significantly associated with this variation. We estimated rates of recombination on two different chromosomes in 205 strains of *D*. *melanogaster*. We also used genome-wide association mapping to identify genetic factors associated with recombination rate variation. We find that recombination rate on the two chromosomes are independent traits. We further find that population-level variation in recombination is mediated by many loci of small effect, and that the genes contributing to variation in recombination rate are outside of the well-characterized meiotic recombination pathway.

## Introduction

Meiotic recombination, the reciprocal exchange of genetic information between homologous chromosomes during meiosis, is necessary for proper chromosome segregation in many organisms [1]. Interestingly, the distribution of meiotic recombination events, or crossovers, varies dramatically in almost all taxa studied to date [2–12]. In addition, crossover frequency varies within and between species and populations in a huge diversity of organisms including humans, chimpanzees, flies, mice, worms, yeast, and many others [3,4,6,8,12–26].

In addition to its role in preserving genomic integrity between generations, recombination is a pivotal force in evolution. Recombination can reduce interference between a genetic variant and the genetic background in which it resides, thereby increasing the efficacy of natural selection [27–29]. Moreover, the exchange of genetic material between homologs creates new allelic combinations and thus contributes to the raw material for the process of evolution. Further highlighting its importance for evolution in general and genome evolution in particular, rates of recombination correlate with numerous genomic features such as the level of DNA polymorphism [30–32], rates of protein evolution [33,34], density of transposable elements [35–38], density of satellite DNA [39,40], and codon bias [41,42].

Given the importance of recombination and the pervasive natural variation in recombination rate, it is perhaps unsurprising that the genetic basis of this variation has been an active area of research for the last decade. With respect to the genetic basis of the distribution of crossover events, the first known determinant of recombination distribution in metazoans was discovered recently [43–45]. This remarkable discovery implicates PRDM9 in determining the locations of meiotic recombination hotspots in both humans and mice. Sequence variation within *Prdm9* also modulates hotspot activity in humans [46]. PRDM9 is a histone methyltransferase that catalyzes histone H3 lysine 4 trimethylation [47]. This rapidly evolving protein [48] was first associated with hybrid sterility in rodents [49], and evidence continues to accumulate that it is a major component of recombination hotspot determination in mammalian systems [46,50–55].

Comparatively less is known in other systems such as Drosophila. Several studies have identified sequence motifs associated with recombination events [7,11,12,56–59], but none have been functionally validated to date. Drosophila lacks PRDM9 [48,58], and perhaps relatedly, also lacks the highly punctate recombination landscape seen in mammals. While in humans up to 80% of recombination events fall in 10–20% of sequence [6], crossover distribution in Drosophila is far less heterogeneous [12,60].

Recent work in mammals has also provided insight into the genetic architecture of global recombination rate. RNF212 has been repeatedly associated with natural variation in recombination rate in several systems including humans [61,62], cattle [63], and Soay sheep [64]. Consistent with a role of this protein in modulating recombination rate, RNF212 is essential for meiotic recombination and has a key role in stabilizing meiosis-specific recombination factors in mice [65]. PRDM9 has also been associated with heritable variation in recombination rate in humans and mice [52,66]. Other mediators of recombination rate include REC8 [63], which is a cohesin that is required for proper chromosome segregation in many organisms [67–69]. In humans, inversion 17q21.31, a 900 kb inversion, is associated with increased recombination and reproductive output in European females [70].

The genetic architecture of recombination rate variation outside of mammals remains poorly understood, even in the model organism *Drosophila melanogaster*. However, it is well-documented that recombination rate is a variable and heritable trait in Drosophila. For instance, classical genetic experiments indicate that the amount of crossing-over as well as the distribution of crossover events can vary among lines of *D*. *melanogaster* [12,13,71,72], suggesting population-level variation in this trait. Additionally, genetic control for crossover rate has been suggested by laboratory selection experiments in which recombination rate itself was successfully subject to artificial selection [73–85]. Finally, changes in recombination rate have been shown to evolve as a correlated response to artificial selection on other characteristics, such as sternopleural bristle number [86], DDT resistance [87], geotaxis [88], and resistance to temperature fluctuations [89], which is again consistent with segregating natural variation in recombination rate. Additionally, the observation that modifiers of recombination rate are commonly associated with variants controlling completely unrelated traits suggests that these modifiers are pervasive in the genome and/or may have pleiotropic effects.

To gain the first insight into the genetic basis of population-level variation in recombination rate in *D*. *melanogaster*, we used an association mapping approach. We favored an unbiased approach in part because *D*. *melanogaster* lacks homologs of the three known determinants of recombination rate in mammals noted above: RNF212, REC8, and PRDM9. We measured recombination rates on both the *3R* and *X* chromosomes in the 205 fully-sequenced inbred lines of the *Drosophila melanogaster* Genetic Reference Panel (DGRP) [90,91] using a two-step crossing scheme. We find nearly 2-fold variation in recombination rate among lines with a standard karyotype. Unexpectedly, we find that recombination rates are uncorrelated between the *X* and *3*^rd^ chromosomes. We leveraged this pervasive population-level variation in recombination rate for genome-wide association (GWA) mapping to identify dominant or semi-dominant variants associated with phenotypic variation in recombination rate on each chromosome. We selected the top 20 most promising candidate genes associated with recombination rate and subjected these candidates to both gene-level and allele-level functional assessment. Our functional assays implicate five highly promising candidates as novel mediators of recombination rate variation in *D*. *melanogaster*: *CG10864*, *CG33970*, *Eip75B*, *lola*, and *Ptp61F*. Our results provide new insight into the scale and scope of population level variation in rates of recombination and more importantly implicate new determinants of natural variation in recombination rate in Drosophila.

## Results

### Robustness of Data

**Fig 1:**
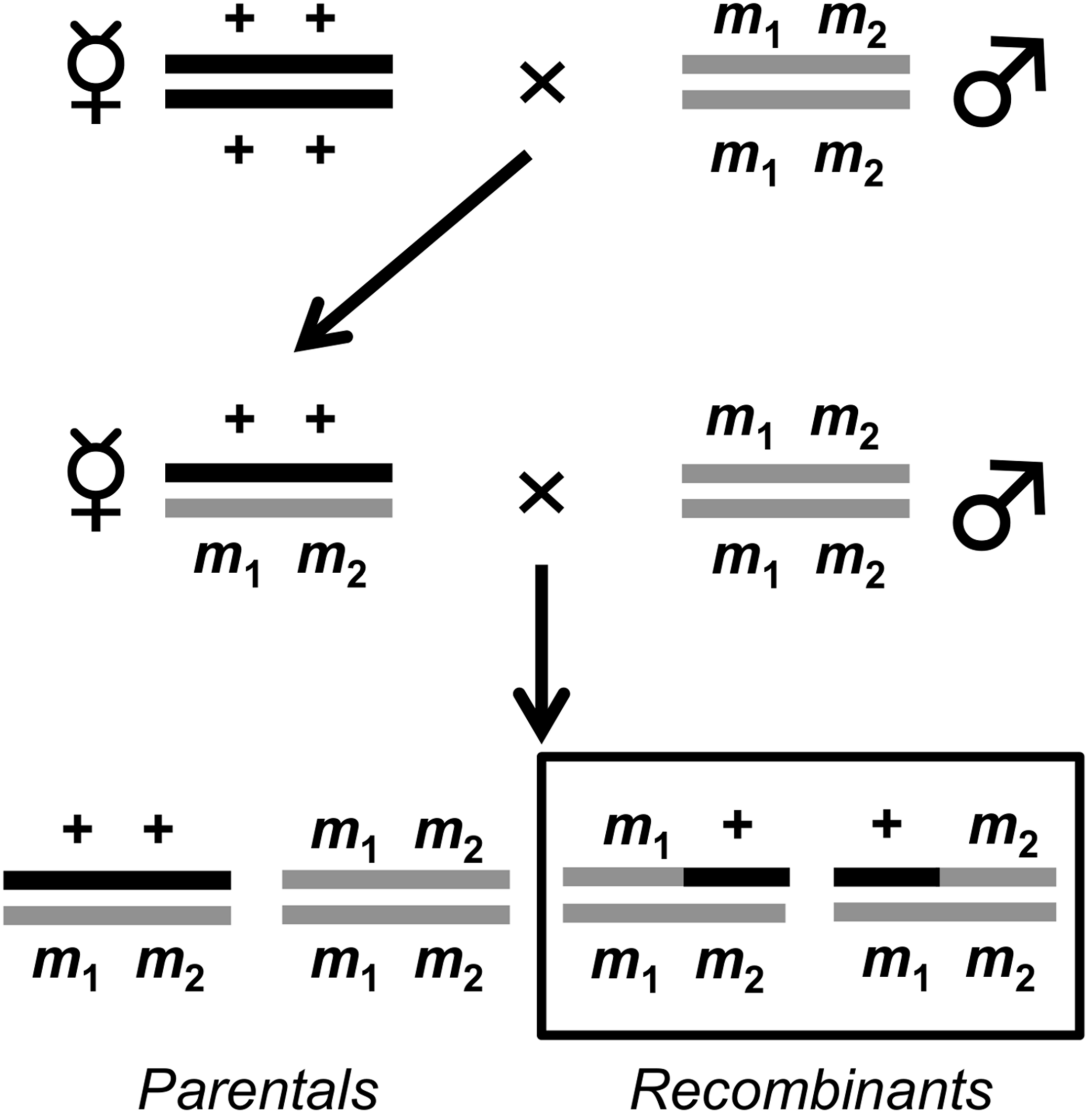
Two step crossing scheme to measure crossover frequency in the DGRP. + + denotes wild-type and *m*_1_ *m*_2_ denotes either the doubly marked *e ro* on chromosome *3R* or doubly marked *y v* on the *X* chromosome. Since males are heterogametic, only one copy of the *y* or *v* marker is needed to display a phenotype. Crossover frequency is calculated by taking the ratio of the total number of recombinants (denoted by black box) to the total number of progeny.

To assay recombination rate variation in the DGRP, we used a classic two-step crossing scheme (Fig 1). We measured recombination rates in two different genomic intervals: the 20.4 cM interval between *ebony* (*e*) and *rough* (*ro*) on chromosome *3R* and the 33 cM interval between *yellow* (*y*) and *vermilion* (*v*) on the *X* chromosome. In total, 506,045 progeny were scored for recombinant phenotypes (217,525 for the *e ro* interval and 288,520 for the *y v* interval). On average, each replicate (there were three replicates per DGRP line per chromosome assay) contained ~368 progeny (for the *e ro* interval) and ~499 progeny (for the *y v* interval). We first verified that our data conformed to expectations under Mendelian inheritance. Deviations from these expectations would be consistent with viability defects associated with the visible markers used in this study. To do so, for each line we compared the number of wild-type progeny to the number of progeny possessing both markers (either *e ro* or *y v*), summing across all three replicates (S1 Table). We also compared the number of recombinant progeny possessing only one marker to the number of recombinant progeny containing only the other marker (either *e +* versus *+ ro* or *y +* versus *+ v*) (S1 Table). The null expectation is a 1:1 ratio for the aforementioned pairs of phenotype classes. We used a Bonferroni correction [92,93] with α = 0.05 to correct for multiple tests. When comparing the ratios of the two non-recombinant haplotypes, we find 15 lines that deviate from the expected 1:1 wild-type: *e ro* ratio (Bonferroni-corrected *P* < 0.03, all comparisons, *G*-test) and 8 lines that deviate from the expected 1:1 wild-type: *y v* ratio (Bonferroni-corrected *P* < 0.03, all comparisons, *G*-test). In all but one case, the deviation is in the direction of a relative excess of wild type flies. Only one line deviated significantly in both intervals (DGRP_819), with more wild-type progeny in both intervals. When comparing the ratios of the two recombinant haplotypes, we find that DGRP_31 deviates significantly from the expected 1:1 *e* +/ + *ro* ratio (Bonferroni-corrected *P* < 0.0001, *G*-test) and that DGRP_819 deviates significantly from the expected 1:1 *y* +/ + *v* ratio (Bonferroni-corrected *P* < 0.0001, *G*-test).

Similarly, we tested for sex ratio unity by comparing the numbers of female and male progeny. There are no deviations from the expected 1:1 male:female ratio in the 205 lines for the *e ro* interval (Bonferroni-corrected *P* > 0.10, all comparisons, *G*-test). For the *y v* interval, only two lines significantly deviate from expectation (DGRP_41 AND DGRP_801) (Bonferroni-corrected *P* < 0.0002, both comparisons, *G*-test), both in the direction of a relative excess of females.

To assess the consequences of possible viability defects associated with our visible markers on recombination rate estimation, we analyzed correlations between viability defects and recombination. That is, to address whether epistatic interactions between our visible markers and DGRP genotype yield viability defects, we analyzed whether the ratios of the number of males vs. females, + + individuals versus *m*_1_ *m*_2_ individuals, or *m*_1_ + individuals versus + *m*_2_ individuals are correlated with our estimates of recombination within the DGRP (S2 Table). Again, each of these ratios should be 1, but could be skewed by viability defects associated with the markers. Our analysis demonstrates that in the *y v* interval, none of these ratios are correlated with our estimates of recombination rate. For the *e ro* interval, we observe a weak but statistically significant correlation between the ratio of wild type progeny to *e ro* progeny and recombination rate. However, no significant correlation is seen between the sex ratio and recombination rate or the ratio of the two classes of recombinants and recombination rate for the *e ro* interval. These data are consistent with weak epistatic interactions between the *e ro* genetic background and wild-type genetic backgrounds that yield viability defects.

Overall, however, our data indicate that our assays for measuring recombination rate largely conform to expectations given Mendelian inheritance. There does not appear to be a large systematic bias towards wild-type chromosomes, indicating that there are no major viability defects associated with any of these mutations alone or in the pairs in which they were used for the current experiment. This confirms previous descriptions of these mutants and their lack of viability defects [94,95]. Our analysis does indicate weak viability effects of the *e ro* background as revealed by epistatic interactions with wild-type genetic backgrounds. As a consequence, the scale and scope of the reported variation in recombination rate may be mis-estimated. Given how weak the viability defects appear to be, we believe any mis-estimation is likely to be small in magnitude.

### Heritable Continuous Variation in Recombination Rate among DGRP Lines

**Fig 2:**
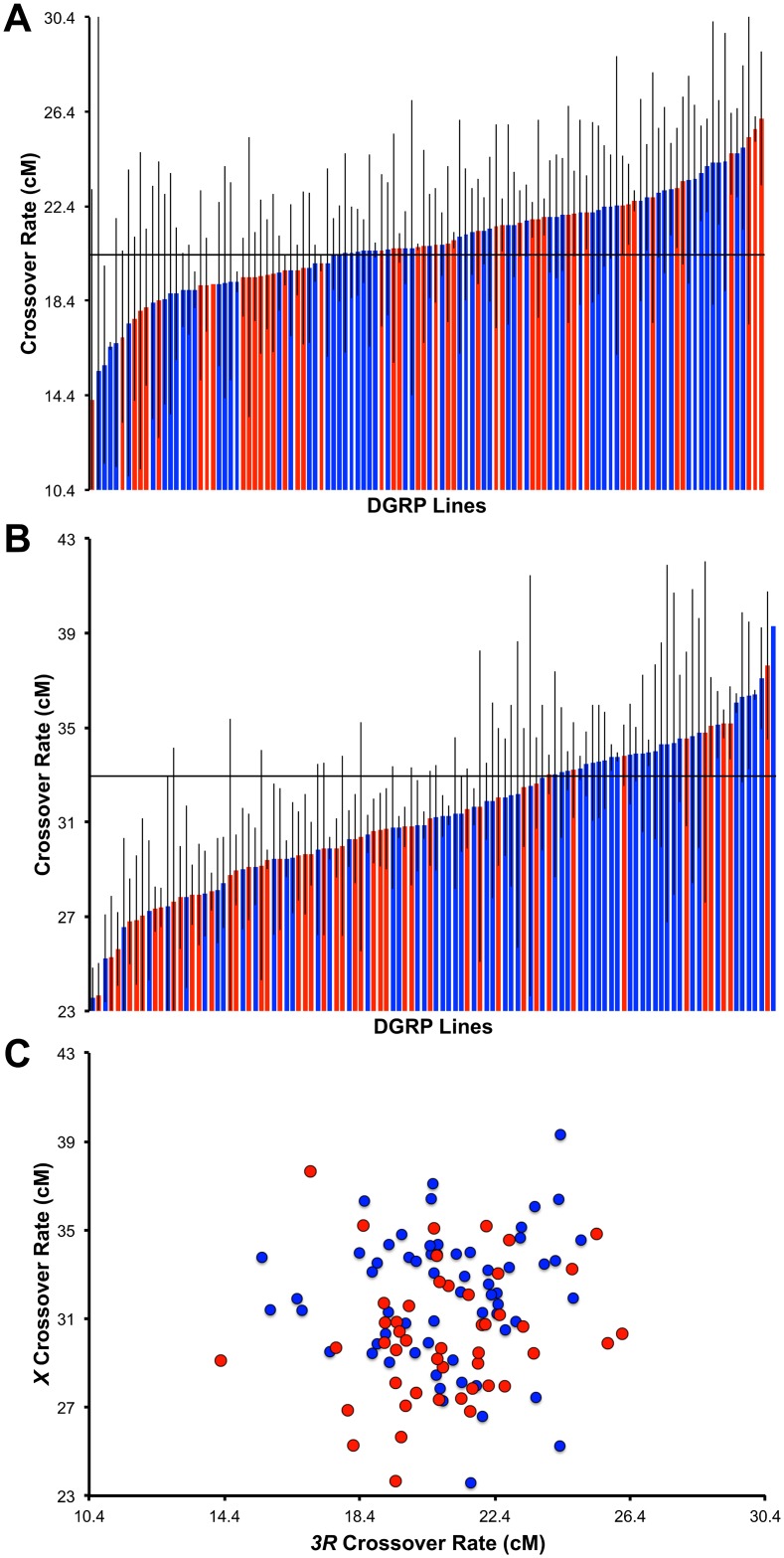
Natural variation in recombination rate. Variation in crossover frequency in the DGRP (standard karyotypes only) in the (A) *e ro* interval on chromosome *3R* and (B) in the *y v* interval on the *X* chromosome. The strains in each panel are ordered by recombination rate. DGRP lines infected by Wolbachia are indicated in blue while DGRP lines not infected by Wolbachia are indicated in red. Grey bars depict standard error. For reference, the reported map distance for the *e ro* interval is 20.4 cM, while the reported map distance for the *y v* interval is 33 cM, indicated by a horizontal line in both graphs. (C) Recombination rate on *3R* as a function of recombination rate on the *X* chromosome (standard karyotypes only).

Following the crossing scheme detailed in the Materials and Methods and in Fig 1, we estimated crossover rate for each DGRP line in the *e ro* and the *y v* intervals (S2 Table; S1A and S1B Fig) for three replicates. These replicates are largely consistent with one another (S3 Table; S2A–S2L Fig). Analyzing only lines with a standard karyotype on all chromosomes (*n* = 112), the average crossover rate for *e ro* is 20.9 ± 0.2 cM (ranging from 14.2 cM to 26.12 cM) (Fig 2A). This agrees well with the published map distance of 20.4 cM [95]. Among these lines, we observe 1.84-fold variation in mean crossover rate. Analyzing only lines with a standard karyotype on all chromosomes, the average crossover rate for *y v* is 31.2 ± 0.3 cM (ranging from 23.6 cM to 39.30 cM) (Fig 2B), compared with the published map distance of 33 cM [94]. Similar to the magnitude of population-level variation in recombination rate on *3R*, here we observe 1.67-fold variation among these lines in mean crossover rate for the *y v* interval.

There is significant genetic variation for crossover rate among lines for both intervals (*F*_*e ro*_ = 1.34, *P*_*e ro*_ = 0.038 and *F*_*y v*_ = 3.00, *P*_*y v*_ < 0.0001, ANOVA). Using only lines with a standard karyotype (112 lines), we estimated broad-sense heritability (*H*^2^) of recombination rate for the *e ro* interval as 0.12 and for *y v* interval as 0.41 (Table 1). These results confirm that recombination rate is a heritable trait and has a genetic component. Interestingly, there is no significant correlation between recombination rates in these two intervals (Spearman’s *ρ* = 0.09, *P* = 0.36; Fig 2C). Consistent with this, a model fitting effects of line, genomic interval, and line-by-interval interaction effects reveals significant interaction effects (*P* < 0.0001, ANOVA, S4 Table), indicating that the magnitude of the difference in recombination frequency between the two loci surveyed varies significantly among lines. These analyses illustrate that recombination rate on chromosome *3R* and chromosome *X*, at least in the way they have been assayed here, are independent traits in this panel of flies.

**Table 1:** Analyses of variance of recombination rate. Results are displayed for both the *e ro* and *y v* intervals (using only lines with standard karyotypes). *H*^2^ denotes broad sense heritability.

Interval	Source	df	Type III SS	MS	F	*P*-value	σ2	*H*^2^
*e ro*	Line	110	0.21	0.0019	1.35	0.035	1.95E-04	0.12
	Error	200	0.29	0.0014			1.14E-03	
*y v*	Line	109	0.33	0.0030	2.95	< 0.001	0.00072	0.41
	Error	204	0.21	0.0010			0.0011	

### Correlation with Other Phenotypes

As a widely-used community resource, the DGRP offers a unique opportunity to examine the relationship between recombination rate and other phenotypes because a variety of phenotypes have been surveyed in this panel. We tested whether crossover rates in the *e ro* or *y v* interval (of lines with standard karyotypes) were correlated with various traits including organismal fitness. While the majority of correlations were weak and not statistically significant, we elaborate on several interesting significant correlations (S23 Table) in S1 Text.

### Interchromosomal Effect

Recombination is suppressed within inverted regions, and recombination elsewhere in the genome increases through what is known as the interchromosomal effect [96,97]. A large number of the DGRP lines are either homozygous or polymorphic for a chromosomal inversion. To test for the interchromosomal effect, we separated lines with inversions from lines with standard karyotypes and tested whether lines that possessed an inversion somewhere in the genome had higher rates of recombination in our surveyed intervals. Lines with inversions have significantly increased rates of recombination in the *y v* interval relative to lines with standard karyotypes (35.1 cM vs. 31.0 cM, *P* < 0.0001, t-test). This trend is echoed in the *e ro* interval (20.9 cM vs. 20.7 cM) but the difference in recombination frequency between standard and inverted karyotypes is not statistically significant (*P* = 0.66, t-test). These results are discussed in the context of previous work in S1 Text.

### Effects of Wolbachia

In the DGRP, 108 lines are infected with *Wolbachia pipientis* [91]. To test for an effect of Wolbachia infection on recombination frequency, we used a linear model (see Materials and Methods) and fit effects of line and Wolbachia infection status for each interval surveyed. Analyzing only lines with standard karyotype, we find there is a significant effect of Wolbachia infection in the *y v* interval (*P* = 0.0003, ANOVA), such that Wolbachia-infected lines have a higher crossover frequency (31.8 cM) than uninfected lines (30.0 cM). No effect of Wolbachia infection was found for the *e ro* interval (*P* = 0.35, ANOVA). Importantly, estimates of heritability are not driven by Wolbachia infection in either interval (S5 Table).

### Genome-Wide Association Analyses

The continuous variation for recombination rate among lines described above (Fig 2A and 2B) suggests that the genetic architecture of this trait is likely complex and regulated by many independent genetic factors. The observed variation in recombination rate in the DGRP motivates our association mapping approach to more finely define the genetic basis of this trait. To identify genetic variants contributing to variation in recombination rate, we performed genome wide association mapping on the mean crossover rates from the DGRP in the *e ro* and *y v* intervals. Note that given the experimental design of our study (Fig 1), we are only able to identify variants that are at least partially dominant in their effects on recombination frequency. Recessive modifiers are not captured in this study, likely yielding underestimates of the scope of natural variation in recombination rate in this system. We did the association mapping in three different ways for each interval because of the inversions segregating in the DGRP and the known effect of inversions on recombination frequency (see [97] for review). Of the inversions segregating in the DGRP, none are on the *X* chromosome. However, 49 lines contained at least one copy of the *C*, *K*, *Mo* or *P* inversion on chromosome arm *3R*; all four of these inversions span at least part of the *e ro* interval used to assay recombination rate [99]. We thus exclude these lines when analyzing recombination rate data for the *3R* interval. The three datasets used for the *3R* analyses were: 1) lines with no inversion on *3R* (n = 156), 2) lines with neither *3R* inversions nor inversion polymorphisms elsewhere in the genome (n = 130), and 3) lines with the standard karyotype (lines lacking inversions; n = 112). The three datasets used for the *X* chromosome analyses were: 1) all lines (n = 205), 2) lines without inversion polymorphisms (n = 152) and 3) lines with a standard karyotype (n = 112).

The statistical model used to infer associations assesses and adjusts for significant associations of both Wolbachia status and inversions. For the *e ro* interval, there is a significant effect of the *NS* inversion (*P* = 0.003, ANOVA; Table 2) on crossover rate in the restricted data set that removes lines with inversions on *3R* and lines with inversion polymorphisms. For the *y v* interval, *Wolbachia* infection is significantly associated with crossover rate in all three of our data sets (*P* < 0.01, all cases, ANOVA; Table 2). Additionally, inversions *t*, *NS*, *K*, and *Mo* are all significantly associated with crossover rate in the *y v* interval (*P* < 0.05, all cases, ANOVA Table 2). These data are summarized in Table 2.

**Table 2:** Analyses of variance of the effects of Wolbachia infection and inversions on recombination rate.

Interval	Data Set	# of DGRP Lines	*P* values					
			Wolbachia Status	*2L*(*t*)	*2R*(*NS*)	*3R*(*P*)	*3R*(*K*)	*3R*(*Mo*)
*e ro*	All lines (with no *3R* inversions)	156	0.17	0.12	0.59	-	-	-
*e ro*	No inversion polymorphisms (and no *3R* inversions)	130	0.31	0.12	**0.003**	-	-	-
*e ro*	Only standard karyotypes	112	0.53	-	-	-	-	-
*y v*	All lines	205	**0.01**	**< 0.0001**	**0.0006**	0.1	**0.002**	**0.001**
*y v*	No inversion polymorphisms	152	**0.006**	**< 0.0001**	**0.002**	0.16	**0.049**	**0.0004**
*y v*	Only standard karyotypes	112	**0.008**	-	-	-	-	-

The full results for all six GWA analyses are presented as supplementary tables (S6–S11 Tables). To generate a list of candidate genes and alleles, we combined the results from the different GWAS for each chromosome interval, using a significance threshold of *P* < 10^−5^. For a Venn diagram displaying overlap among the different data sets, see S3 Fig. We tested whether the distribution of these associated variants was significantly different from the null expectation of a uniform distribution across chromosomes (as a function of the number of polymorphisms on each chromosome). Using lines with standard karyotypes, we find that the distribution of associated variants is significantly different from the distribution of variants in the genome for both intervals (*P* < 0.02, both comparisons, *G*-tests). It appears that in both intervals, there is an enrichment of associated variants on chromosome *2R* (*e ro*: 63 versus 33; *y v*: 29 versus 16; observed versus expected).

**Fig 3:**
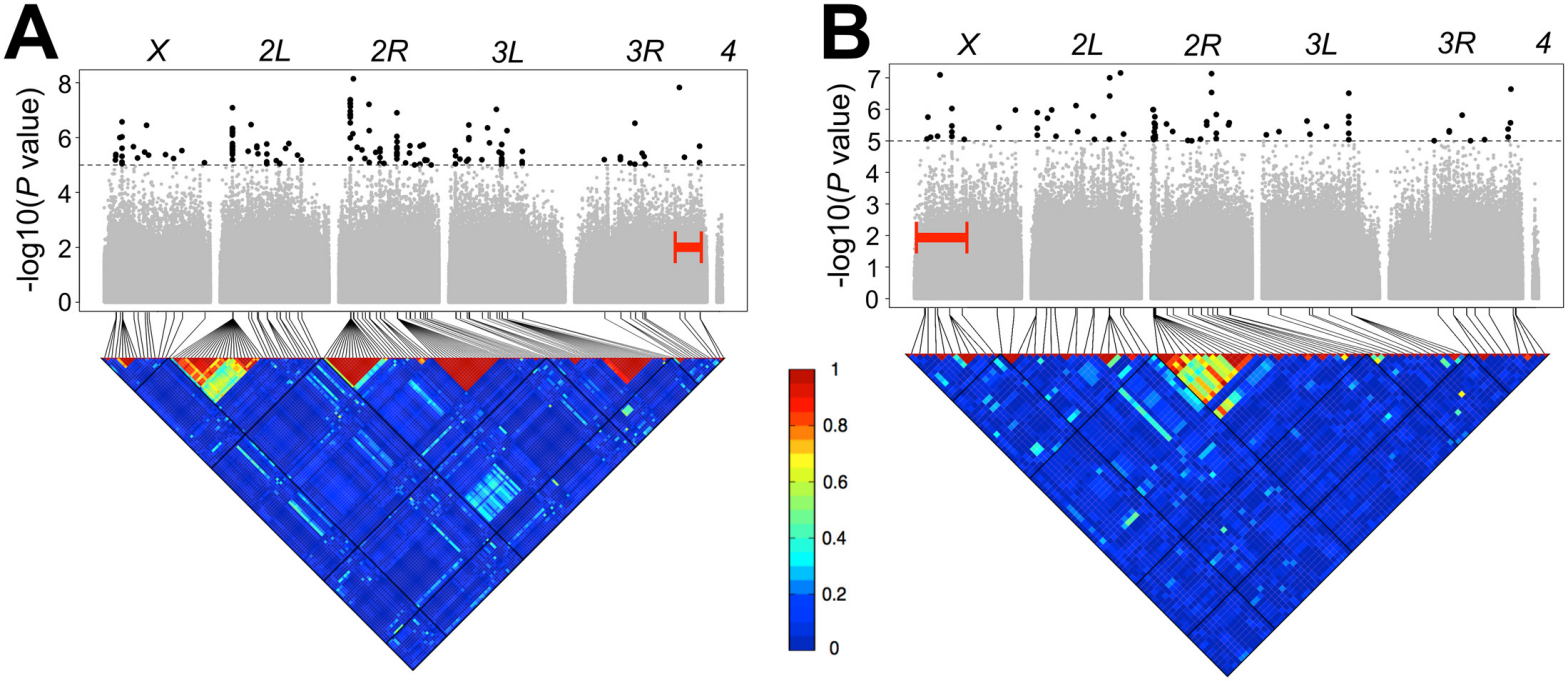
Genome-wide association analyses. Manhattan plots and accompanying linkage disequilibrium heat maps are depicted for the (A) *e ro* interval and (B) *y v* interval for lines with standard karyotypes. A significance threshold of *P* ≤10^−5^ is denoted for Manhattan plots. Each point is a tested genetic variant in the DGRP and points above this threshold (in black and enlarged to aid in visualization) indicate significantly associating variants. Additionally, the surveyed interval for each chromosome (either *e ro* or *y v*) is bracketed in red. The triangular heat map displays the amount of linkage disequilibrium (LD, measured here as *r*^2^) between variants. Each major chromosome is depicted. Red denotes complete LD and blue denotes absence of LD.

For the *e ro* interval, the three GWAS yielded a combined total of 688 unique variants at a nominal significance threshold of *P* < 10^−5^. For the *y v* interval, combining results from all three GWA analyses, we identified 160 unique variants at a nominal significance threshold of *P* < 10^−5^. A description of types and locations of these variants is included in S12 Table. There were no variants that overlapped between the two intervals, consistent with the lack of correlation between the two traits. However different variants in the same gene (see below) were shared between the associations found in the two intervals. Variants in 359 genes were implicated as potential candidates from the three *e ro* GWAS, and variants in 111 genes were associated with recombination rate variation in the *y v* GWAS. There is very little overlap between these gene lists; a total of fifteen genes showed overlapping (gene-level) associations between the *e ro* and *y v* GWAS (*bab1*, *bun*, *CG4440*, *CG5953*, *CG31817*, *CG32521*, *CR44199*, *dnr1*, *dpr6*, *Eip63E*, *Eip75B*, *Ptp61F*, *Sec16*, *Shroom*, and *SNF4Agamma*). The effect sizes for these variants were moderate, averaging ~2.32 cM for both intervals (S4A and S4B Fig). Fig 3A and 3B displays the Manhattan plots and linkage disequilibrium plots for both intervals for the lines with standard karyotypes while S5 and S6 Figs display the same information for the other data sets analyzed.

### Candidate Genes

We sought to functionally assess a subset of the genes identified by our association mapping. We used several criteria to refine our list of candidate associations to a tractable set of 20 candidate genes. First, we restricted our focus to protein-coding genes harboring significantly associated genetic variants. We then integrated the *P*-value of the association, effect size, and the number of GWAS the gene was implicated in on either or both chromosomes to refine our list of putative candidates. We narrowed our list further by limiting ourselves to genes with documented expression in the ovaries [99–103]. Our final candidate gene list (Table 3) includes eleven genes from the *e ro* GWA, five genes from the *y v* GWA and four genes that were found in both. There was more than one significantly associated genetic variant in 8 of our 20 candidate genes (*CG1273*, *CG4440*, *CG7196*, *dpr6*, *Eip75B*, *jing*, *Ptp61F* and *Ubx*) with *jing* and *Ptp61F* having the most significantly associated variants (17 and 13 respectively). The full list of variants within these genes and associated *P*-values are listed in S13 Table and the genotypes of each DGRP line at these variants are listed in S14 Table.

**Table 3:** Summary of Candidate Genes identified during GWAS and selected for functional assessment. We report the number of GWAS the gene was implicated in out of six total. modENCODE expression [100,102,103] is listed as mRNA signal in ovaries (i.e. virgin mRNA signal / mated mRNA signal). FlyAtlas expression [99] is listed as mRNA signal in ovaries as well as whole flies (i.e. ovary mRNA signal / whole fly mRNA signal). mRNA expression from early ovarian tissues [101] is listed as either expression in only ‘early’ meiosis (germaria to stage 3), ‘late’ meiosis (remaining ovarioles), ‘both’ phases, or significant differential expression (‘DE’) between the early and late.

Candidate Gene	GWAS	Number of GWAS	Lowest *P*-value	Largest Effect Size	modENCODE Expression [100,102,103]	FlyAtlas Expression [99]	Early Ovarian Tissues [101]
*alph*	*y v*	1	2.29E-07	3.44	167 / 181	207 / 222	Both
*bru-2*	*e ro*	1	1.14E-05	1.57	2 / 2	2 / 9	Both
*cdi*	*e ro*	1	9.22E-06	1.63	35 / 26	6/60	Both
*CG1273*	*e ro*	1	3.47E-07	2.61	-	3 / 12	Both
*CG4440*	*e ro* & *y v*	6	1.10E-08	3.00	- / 1	2 / 46	Early
*CG7196*	*e ro*	3	8.00E-07	3.01	-	2 / 80	Both
*CG9650*	*e ro*	1	5.54E-06	2.34	-	12 / 9	Both
*CG10864*	*e ro*	1	3.72E-06	2.39	-	5 / 6	Both
*CG15365*	*e ro*	1	3.55E-07	2.61	1 / 1	6 / 8	Both
*CG33970*	*y v*	1	5.66E-05	2.81	1 / 1	56 / 366	Both
*dpr6*	*e ro* & *y v*	4	9.37E-08	3.09	16 / 15	20 / 13	Both
*Eip75B*	*e ro* & *y v*	2	5.98E-06	2.69	4 / 3	115 / 68	Both
*grp*	*e ro*	1	3.31E-06	-1.10	122 / 110	2137 / 1069	DE
*jing*	*e ro*	3	4.17E-08	2.79	16 / 17	72 / 26	Both
*lola*	*e ro*	1	6.13E-08	2.76	130 / 143	112 / 95	DE
*MESR3*	*y v*	1	5.90E-06	2.28	16 / 12	197 / 236	Both
*Oaz*	*y v*	1	8.84E-06	2.68	-	1 / 1	Both
*pk*	*e ro*	1	7.20E-09	2.92	-	6 / 13	DE
*Ptp61F*	*e ro* & *y v*	3	3.36E-08	3.15	91 / 85	928 / 359	DE
*Ubx*	*y v*	1	4.82E-06	1.53	-	1 / 13	Both

### Functional Assessment

**Fig 4:**
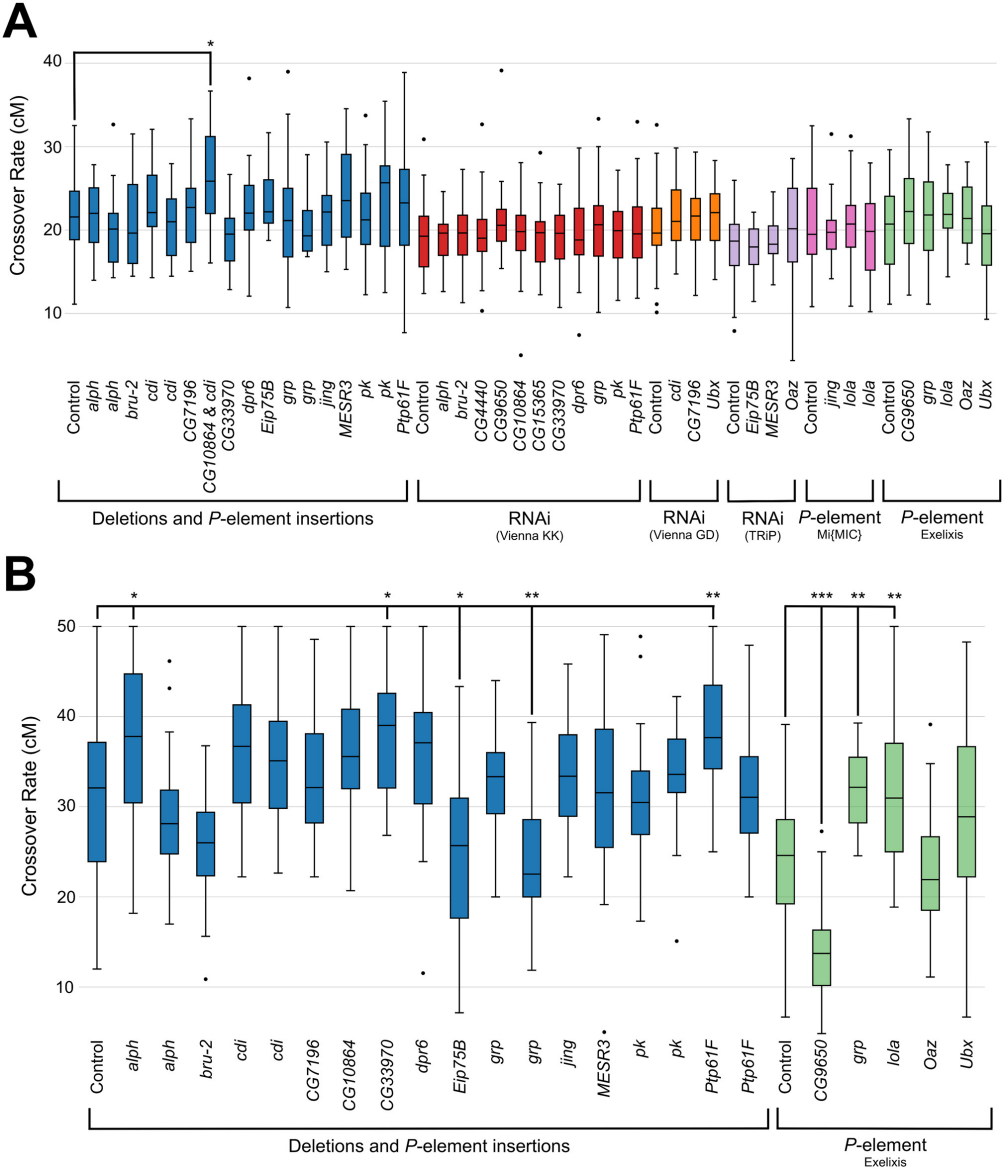
Functional assessment of candidate genes. Recombination rate of *P*-element insertions, chromosomal deletions and RNAi lines assayed in the (A) *e ro* interval and (B) *y v* interval. Experimental strains are compared to the control strain (first boxplot in each bracketed set). Boxplots show first to third quartiles with whiskers extending to the smallest and largest nonoutliers. The median is indicated by a black line in each box. * indicates a *P* < 0.05, ** indicates *P* < 0.01 and *** indicates *P* < 0.001 (via Dunnett’s test of recombination proportions).

If these identified candidate genes mediate recombination rate in some way, we expect that perturbing these genes will affect recombination rate. We used both mutant analysis and RNAi to either knock out or knock down expression of each of these genes, and compared recombination rate in the knock out/down lines to an appropriate genetic background control. We measured recombination rate in the *e ro* and *y v* intervals for available mutants and RNAi lines for all 20 candidate genes in the same way as described earlier. We used a combination of *P*-element insertions, chromosomal deletions, as well as any available RNAi lines. For the RNAi experiments, we used a *nanos GAL4* driver, which should target the effects of knockdown to oogenesis. For assessment using the *e ro* markers, the only line tested that produced a significant difference from control line was a deletion line, *Df(3R)ED2* (*P* = 0.004, Dunnett’s test) (Fig 4A; S15 Table); this line shows a significant increase in recombination frequency relative to the genetic background control. This deletion encompasses 71 full genes and part of 1 additional gene, including two of our candidate genes: *cdi* and *CG10864*. It should also be noted that this deletion is on chromosome *3R*, spanning the cytological region 91A5 to 91F1 (for reference *e* is at 93C7-93D1 an*d ro* is at 97D4-97D5). Using the *y v* markers, seven lines tested show a significant deviation in recombination frequency relative to the appropriate control (Fig 4B; S16 Table). These included *alph*, *CG9650*, *CG33970*, *Eip75B*, *grp*, *lola*, and *Ptp61F* (*P* < 0.05, all comparisons, Dunnett’s test). *Eip7B*, and *CG9650* showed a decrease in recombination relative to the control while *alph*, *CG33970*, *Eip75B*, *lola*, and *Ptp61F* showed an increase in recombination relative to the control. Interestingly, one *P*-element insertion in *grp* showed a significant increase of recombination while a different *P*-element insertion in *grp* showed a significant decrease of recombination.

### Expression Analysis

**Fig 5:**
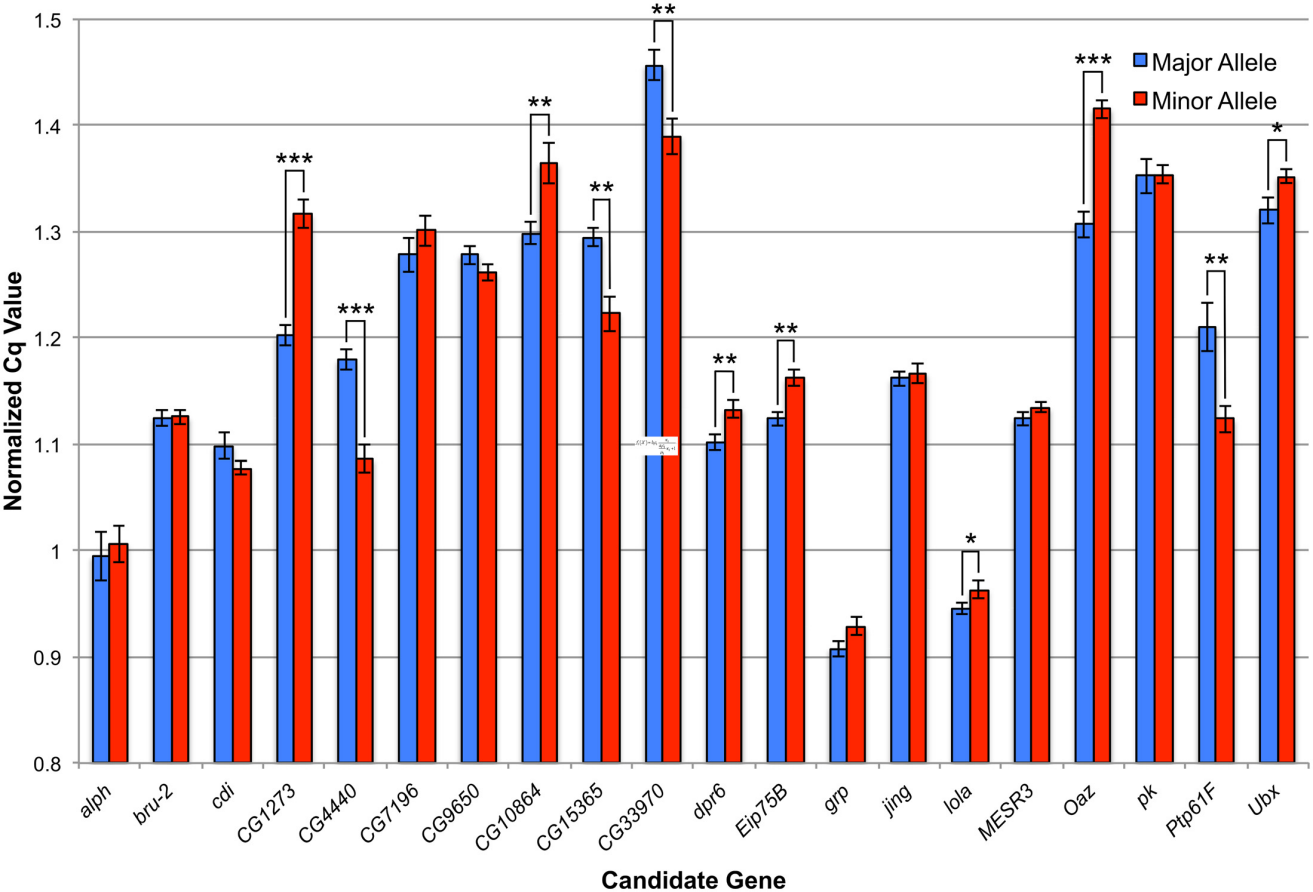
Assessment of expression difference in ovaries via qPCR. For each candidate gene, the normalized average expression of three lines with the major allele (blue bars) and three lines with the minor allele (red bars) are shown. Error bars denote standard error. * indicates a *P* < 0.05, ** indicates *P* < 0.01 and *** indicates *P* < 0.001 (via a student’s t-test).

While the mutant/RNAi analysis provides insight into whether the candidate genes function in some way to mediate recombination, we also wanted to test whether these candidate genes show significant differences at the allelic level. We hypothesized that the effects of these genes on recombination rate were mediated by expression level differences and thus tested for differences in gene expression in ovaries between allelic variants of our 20 candidate genes. We measured gene expression as mRNA abundance using quantitative RT-PCR (qPCR). For each of our twenty candidate genes, we selected three DGRP lines containing the major allele and three lines containing the minor allele (S17 Table). For candidate genes that had that multiple significantly associated variants, all attempts were made to include lines in which all minor alleles were present. The genotypes of these lines at the gene surveyed can found in S18 Table. Once a line was selected to assess a candidate gene, it was not used to assess another candidate gene. RNA was extracted from dissected ovaries from virgin DGRP females. The qPCR data (normalized to *GAPDH*) reveal significant differential expression for 11 of our 20 candidate genes (Fig 5; S19 Table). DGRP lines with the major alleles of *CG4440*, *CG15365*, *CG33970*, and *Ptp61F* (*P* < 0.003, all comparisons, t-test) display higher expression levels than lines with the minor alleles. Conversely, DGRP lines with the major alleles of *CG1273*, *CG10864*, *dpr6*, *Eip75B*, *lola*, *Oaz*, and *Ubx* (*P* < 0.05, all comparisons, t-test) display lower expression levels than lines with the minor alleles. It should be noted for variants in these eleven candidate genes, all minor alleles are associated with reduced rates of recombination. Comparisons of un-normalized data (given potential concern over unstable housekeeping gene expression [104,105]) largely confirm these results (S20 Table; S7 Fig).

## Discussion

### Population Level Variation in Recombination Rate

Here we report the largest population-level survey of recombination rate variation in Drosophila to date. We find significant genetic variation for recombination rate in this North American population of *D*. *melanogaster* for two independent genomic intervals. At the broadest scope, these data are consistent with previous work from other systems. Indeed, a wealth of data indicate that recombination rate varies between and within populations in species such as Drosophila [12,13], mice [23], and humans [5,20,51,106].

The magnitude of population-level variation in recombination rate exposed by our survey is comparable to what has been previously shown in *D*. *melanogaster*. For instance, we observe 1.67 fold-variation for the *y v* interval, and previous work in this interval shows ~1.2-fold variation [13,72]. Other genomic regions in Drosophila consistently show 1–2 fold variation in crossover frequency among strains [13]. Although measured with a different approach, work from heterogeneous stock mice indicates that crossover frequency varies ~2-fold in both males and females [23]. Work from cattle indicates that average genome-wide recombination rate varies ~1.7 fold in males [63], which aligns well with our survey. Similarly, humans show ~2-fold variation in crossover frequency in both males and females [62,107].

It should be noted that the ~2 fold variation in recombination frequency that we report above is biased downward and is not truly reflective of segregating natural variation in recombination rate in Drosophila. When we include lines with inversions, which clearly segregate in natural populations, we see a much greater span in recombination rates in the DGRP: 5.2-fold for the *e ro* interval (excepting lines with an inversion on *3R*) and 3.5-fold for the *y v* interval. This range of variation in recombination frequency is remarkable, nearly doubling previous estimates from Drosophila, mouse and humans. However, it also bears mentioning that we cannot exclude that our estimates of recombination may be biased by the weak viability effects associated with our visible markers (see above).

### Lack of Correlation between Rates of Crossing over on *3R* and *X*

Our results indicate that recombination rate at the two intervals surveyed are uncorrelated in the DGRP. It is certainly possible that the weaker genetic component of phenotypic variation in recombination rate in the *e ro* interval as compared to the genetic component of phenotypic variation in recombination rate in the *y v* interval is driving the lack of correlation between recombination rates in the two intervals. In contrast to what we observe here, previous work in humans showed a significant positive correlation between the number of maternal recombination events on individual chromosomes and the number of maternal recombination events in the remaining genome complement for 20 out of 23 chromosomes, as well as a strong, significant correlation for the first eight chromosomes compared to chromosomes nine through twenty-two and the *X* chromosome [3]. Other work in Drosophila is suggestive that two lines with less crossing over in one interval relative to four other lines generally had less crossing over in other intervals relative to the same four lines [13], though this is anecdotal at best. The putative difference between Drosophila and humans with regard to correlations in recombination rates across chromosomes is interesting, and may point to different genetic architectures of this trait in these systems. Certainly, the molecular mechanics of meiotic recombination have diverged markedly between humans and Drosophila (e.g. [108]) and the recombinational landscapes in humans and flies are qualitatively different as well.

### Heritability

Previous work has estimated heritability for recombination rate in many different species. While estimates of heritability are necessarily population-specific, mammalian estimates encompass a wide range, from as small as 0.14 [109] and 0.30 [110] in humans to as large as 0.46 in mice [23]. In maize, heritability of recombination frequency is considerably higher (broad sense heritability 0.21–0.69; [111]). Insects show a wide range as well, with estimates of narrow sense heritability of recombination rate ranging from 0.16 in Tribolium [112] to 0.27–0.49 in grasshoppers [113]. Early estimates of narrow sense heritability of recombination frequency in Drosophila based on parent-offspring regression are comparable to ours (0.09–0.38; [114]), and selection based approaches yield a narrow sense heritability of 0.12 [79]. That estimates of heritability of recombination are low indicates that much of the observed variation in recombination frequency cannot be ascribed to genetic differences along lines. This is consistent with the remarkable phenotypic plasticity in recombination frequency in Drosophila, evidenced in response to temperature [115–122], maternal age [72,115–117,123–133], nutrition [126,127], parasite pressure [134] and other environmental factors. This phenotypic plasticity could also drive the lower than expected correlations between replicates observed in this experiment (see S3 Table) and also reduce heritability.

### Effects of *Wolbachia pipientis* Infection

*Wolbachia pipientis* is a common endosymbiont that infects the reproductive tissues of many arthropods [135]. Evidence indicates that over 40% of arthropods are infected with *W*. *pipientis* [136–138]. Approximately 29% of Drosophila stocks from Bloomington Drosophila Stock Center [139] are infected, along with 76% of the *Drosophila* Population Genomics Project (n = 116) [140]. In the DGRP, 108 of 205 (53%) lines are infected with *W*. *pipientis* [91]. In Drosophila, there is clear infection in the ovaries [141,142] and infection has been shown to reduce egg production [143].

Interestingly, we see a significant association between Wolbachia infection and crossover rates in the *y v* interval but not in the *e ro* interval. This discrepancy between the two intervals surveyed is difficult to explain, and merits further investigation. More curious yet is the contrast with previous results. It has been shown that Wolbachia infection has no effect on rates of crossing over in the *w ct* interval (18.5 cM) in the laboratory wild-type strain Canton S [144]. The *w ct* interval is actually within the *y v* interval surveyed in this study, so the discrepancy between the two studies is puzzling. It may be that the effect of Wolbachia infection on recombination frequency is sufficiently minor that the previous study, using a single genetic background and smaller sample sizes than the present study, was underpowered to detect this small effect (an average increase of 1.8 cM associated with Wolbachia infection in our study). Our results, coupled with previous findings, suggest that W. *pipientis* might have differential effects on recombination frequencies in different parts of the genome. Testing explicitly for this heterogeneity will be a topic of future exploration. In the future, it will also be interesting to see if infecting DGRP lines with Wolbachia causes an increase of crossover rates and if curing DGRP lines via tetracycline yields a corresponding decrease in crossover rates.

### Candidate Genes

The DGRP allows us to couple phenotypic variation with genetic variation such that the genetic basis of complex traits of interest can be dissected. One benefit of this association mapping approach is that it is unbiased, which means that new genes, outside of known pathways playing a role in the phenotype of interest, can be identified. For example, a recent study using the DGRP dissecting the genetic architecture of abdominal pigmentation yielded associations with several variants in the known pigmentation pathway but importantly, also functionally validated seventeen out of twenty-eight candidate genes that had not been previously associated with pigmentation [145]. Because nothing was known regarding the genetic basis of population-level variation in recombination rate in Drosophila and because Drosophila lacks homologs of all genes associated with recombination rate variation in other systems, we were eager to leverage this unbiased approach to gain novel insight into the genetic architecture of this fundamentally important trait.

Consistent with the power of GWAS to uncover novel genes associated with phenotypic variation, our top candidate genes significantly associated with recombination rate variation contain genes outside of the meiotic recombination pathways, which have been characterized in exquisite detail (see [146] for review). Among the top 20 candidates for functional assessment, seven are computationally predicted genes that have no clearly defined biological function or human orthologs. Interestingly, four of our candidate genes have Cys_2_His_2_ zinc fingers (*CG9650*, *jing*, *lola*, and *Oaz*). This is particularly intriguing due to the link between the zinc-finger domain containing PRDM9 and hotspot determination, and it is tempting to speculate that these proteins bind to DNA and designate crossover sites in a way that is vaguely reminiscent of the role of PRDM9 in mammalian recombination [43–45]. Moreover, the *D*. *pseudoobscura* ortholog of *Oaz*, *GA14502*, was previously identified as a possible candidate gene involved in recombination as the frequency of its zinc finger binding motif was significantly negatively associated with recombination on a broad scale [58]. Consistent with a role for zinc-finger DNA binding in Drosophila recombination, Trem, which also contains zinc fingers, was recently shown to be necessary along with Mei-W68 and Mei-P22 for the formation of double-strand breaks in Drosophila [147].

We chose two methods for functional assessment of our candidate genes. The first method is a gene-level approach and asks whether perturbation of candidate genes perturbs recombination frequencies. To complement this approach, we also compared expression levels of the different alleles in these candidate genes using qPCR. Significant differential expression of the major versus minor alleles of our candidate genes in the ovaries would be consistent with gene expression differences underlying differences in rates of crossing over.

Overall, there were 5 genes (*bru-2*, *CG4440*, *jing*, *MESR3*, and *pk*) which showed neither a change in recombination frequency in the *e ro* or *y v* intervals when perturbed nor a difference in expression level between the major and minor allelic variants. However, lack of functional confirmation does not imply that a candidate gene has no role in modulating recombination rate in Drosophila. Indeed, validation of candidate genes is challenging. The effect sizes of the genetic variants are moderate at best (S4A and S4B Fig), making detection of these changes quite difficult in the absence of very large sample sizes. Additionally, recombination rate variation is likely to be a polygenic trait [77,78], and our results confirm this. Further, it has been suggested that in many quantitative traits within the DGRP, there is pervasive epistasis [148,149]. Epistatic interactions may similarly contribute to recombination rate variation in Drosophila. Consistent with this is the observation that for one *P*-element insertion of *grp*, there is an increase in recombination relative to the appropriate background and a decrease in recombination rate for another *P*-element insertion (though we note that this observation is also consistent with variation in allelic effects at a single locus if the two *P*-elements were inserted into different locations). Finally, the process of recombination is likely to be highly buffered, and one could hypothesize that there is redundancy for maintaining the number of crossovers required. It is also possible that these statistical associations are false positives due to our lenient *P*-value.

However, integrating across both the gene- and allele-level functional analysis, we find five high quality candidate genes for further investigation. These genes show significant perturbations in recombination frequency relative to the appropriate genetic background control in addition to differential expression specifically in ovaries between allelic variants at these loci. These were *CG10864*, *CG33970*, *Eip75B*, *lola*, and *Ptp61F*. Two of these (*Eip75B* and *Ptp61F*) were identified in GWAS in both the *e ro* and *y v* interval.

*CG10864* is involved in potassium channel function [150]. In humans, another protein involved in potassium channel function, KCNQ1, has been shown to somatically imprint regions of the genome with higher rates of recombination [151]. While imprinting appears to be less common in Drosophila females [152], it is unclear if CG10864 is participating in a similar role as compared to KCNQ1.

*CG33970* is predicted to be involved with ATP binding and transporter activity [98]. A direct link between ATP binding and meiotic recombination has yet to be shown, but there have been some hints of connections in the literature. For example, mutations in the ATP-binding domain of *RecA* [153] in *Escherichia coli*, *DMC1* [154], *Rad51* and *Rad55* in yeast [155,156] and *XRCC3* in humans [157] cause defects in homologous recombination and meiosis. While speculative, this gives credence to the idea that the putative ATP-binding ability of *CG33970* may contribute to meiotic recombination. Further work is aimed at dissecting this link.

*Eip75B* (*Ecdysone-induced protein 75B*) is involved in mediating ecdysone signaling, a steroid hormone. Defective ecdysone signaling affects the early germarium, causing defects with meiotic entry [158]. Interestingly, ecdysone signaling is important for female fertility but not for male fertility [159–161]. Drosophila males do not undergo meiotic recombination [162,163]. It remains to be seen whether the connection between recombination, fertility and ecdysone signaling is merely coincidence; however, the role of *Eip75B* in oogenesis makes it a particularly exciting candidate for further work.

*lola*, or *longitudinals lacking*, is BTB zinc finger-containing transcription factor that is required for axon growth and guidance [164,165]. As noted above, DNA binding ability along with zinc fingers is exciting as a possible link with recombination. The predicted human ortholog, *ZBTB46* or *BZEL*, was shown to repress a desumoylase [166]. Sumoylation has been linked to DNA repair [167] and therefore it is possible that *lola* is involved in early processes that could ultimately lead to crossover formation.

*Ptp61F* (*Protein tyrosine phosphatase 61F*) is a member of the protein tyrosine phosphatase family. *Ptp61F* is an induced antagonist of the JAK/STAT pathway [168,169] and has been directly implicated in oogenesis [170]. In the female germline, expression of *Ptp61F* is targeted to the nucleus and cytoplasmic organelles [171] and this gene is required for normal female fecundity [172]. Tentative links between *Ptp61F* and DNA damage can be made in mammals; *Ptp61F* is the Drosophila homolog of human *PTP1B* and knockout *PTP1B* mice show a higher sensitivity to irradiation and an upregulation of many genes in the DNA excision/repair pathway [173]. Homologous recombination, base excision repair, and nucleotide excision repair are the primary pathways by with DNA damage are repaired in Drosophila. While the role for *Ptp61F* in meiotic recombination is not obvious, the clear function of this gene in oogenesis coupled with its tentative connection to DNA damage repair is promising.

### Conclusion

In conclusion, we have quantified the extent of recombination rate variation in a natural population of *D*. *melanogaster* and have shown that genetic background significantly drives phenotypic variation in this critically important phenotype. The magnitude of observed phenotypic variation in recombination rate is large, with almost 2-fold variation present in each genomic interval analyzed. We demonstrate that inversions play a large role in mediating rates of recombination, indicative of the interchromosomal effect, and provide the first evidence that Wolbachia infection can significantly increase rates of recombination. Through our GWA approach, we show that recombination rate is a highly polygenic trait, with many genetic factors of small effect associating with phenotypic variation. We show that a subset of our candidate genes (*CG10864*, *CG33970*, *Eip75B*, *lola*, and *Ptp61F*) play putative roles in modulating recombination rate variation in Drosophila through both gene-level and expression-level functional assessment. Future work will be aimed at determining the role of these candidate genes in the molecular process of recombination.

## Materials and Methods

### Fly Stocks

The *Drosophila* Genetic Reference Panel is a collection of 205 fully-sequenced inbred lines [90,91]. Mated, gravid *Drosophila melanogaster* females were originally collected in Raleigh, NC, USA in 2003. Their progeny were subjected to 20 generations of full-sibling matings. The resulting inbred lines were then fully sequenced. A total of 4,853,802 single nucleotide polymorphisms (SNPs) and 1,296,080 non-SNP variants were identified among these lines [91].

To assay recombination rate, we took advantage of visible, recessive markers in *D*. *melanogaster*. To measure recombination rates on the *3R* chromosome, we used a strain marked with *ebony* (*e*^*4*^) and *rough* (*ro*^*1*^); these markers are 20.4 cM apart [95]. To measure recombination on the *X* chromosome, we used a strain marked with *yellow* (*y*^*1*^) and *vermillion* (*v*^*1*^); these markers are 33 cM apart [94]. These markers were chosen to examine due to the genetic distance between them, ease of scoring and also their apparent lack of viability defects [94,95]. Each of the doubly marked chromosomes was substituted into a wild-type isogenic Samarkand genetic background, free of *P*-elements [174], to allow for continuity between assays and to minimize marker genetic background effects.

### Recombination Rate Assay

To assay recombination rate variation in the DGRP, we used a classic two-step crossing scheme (Fig 1). All crosses were executed at 25°C with a 12:12 hour light:dark cycle on standard media using virgin females aged roughly 24 hours. We conducted three replicate assays for each interval (either *e ro* or *y v*). For each replicate, all 205 lines were crossed simultaneously to avoid conflating block effects with variation among lines. This yielded three replicate estimates of recombination frequency per line per interval. For the first cross, ten virgin females from every DGRP line were crossed to ten doubly-marked males (either *e ro* or *y v*) in eight ounce bottles. Males and females were allowed to mate for five days, after which all adults were cleared from the bottles. F_1_ females resulting from this cross are doubly heterozygous; these females are the individuals in which recombination is occurring. To uncover these recombination events we backcross F_1_ females to doubly-marked males. For this second cross, twenty heterozygous virgin females were collected and backcrossed to twenty doubly-marked males. Males and females were allowed to mate for five days, after which all adults were cleared from the bottles. After eighteen days, BC_1_ progeny were collected, frozen, and scored for sex and for visible phenotypes. Previous work in our lab has demonstrated that freezing flies has no effect on the visible markers we scored. Recombinant progeny were then identified as having only one visible marker (*m*_1_ + or + *m*_2_). For each replicate, recombination rates were estimated by taking the ratio of recombinant progeny to the total number of progeny. Double crossovers cannot be recovered with this assay, so our estimates of recombination frequency are likely to be biased downwards slightly. The estimated recombination for a given strain and interval was calculated as the average across the three replicates.

### Inversions

Freeze 2 of the DGRP contains information relating to 16 segregating autosomal inversions verified by cytological methods [91]. We therefore performed association mapping in three different ways for each interval. The *X* chromosome (in this population of flies) lacks inversions while 49 lines contain an inversion on chromosome arm *3R* which spans at least part of the *e ro* interval used to assay recombination rate [98]. We thus completely exclude these lines when analyzing recombination rate data for the *3R* interval. The three datasets used for the *3R* analyses were: 1) lines with no inversion on *3R* (n = 156), 2) lines with neither *3R* inversions nor inversion polymorphisms elsewhere in the genome (n = 130), and 3) lines with the standard karyotype (n = 112). The three datasets used for the *X* chromosome analyses were: 1) all lines (n = 205), 2) lines without inversion polymorphisms (n = 152) and 3) lines with a standard karyotype (n = 112).

### Statistical and Quantitative Genetic Analyses

To estimate the broad-sense heritability (*H*^2^) of recombination rate, we used an ANOVA framework on line means (the average across the three replicates for each line for each interval). The ANOVA followed the form of *Y* = *μ* + *L* + *ϵ* for each chromosome assayed where *Y* is recombination rate, *μ* is the overall mean, *L* is the random effect of line and *ϵ* is the residual. Additionally, we ran a similar ANOVA, adding the genomic region as a fixed factor, to test for a significant interaction between line and genomic region. That ANOVA followed the form of *Y* = *μ* + *L* + *R* + *L* × *R* + *ϵ*, with the terms the same as above and *R* is the genomic region assayed. To estimate *H*^2^, we follow the formula *H*^2^ = *σ*^2^_*L*_ / (*σ*^2^_*L*_ + *σ*^2^_*ϵ*_) where *σ*^2^_*L*_ is the variance component among lines and *σ*^2^_*ϵ*_ is the residual variance or variance component attributed to error. The variance components were calculated using REML. All *H*^2^ estimates were calculated using R Statistical Software, v3.2.1 and RStudio v0.99.467.

To test for a significant effect of Wolbachia infection, we used an ANOVA framework as well. The ANOVA follows the form *Y* = *μ* + *W* + *ϵ* for each chromosome assayed where *Y* is recombination rate (measured in cM), *μ* is the overall mean, *W* is fixed effect of Wolbachia infection status and *ϵ* is the residual, including all individual measurements.

### Genome-Wide Association

To identify genetic variants that are associated with differences in mean crossover number in two different intervals of the Drosophila genome, we performed a GWAS using the established web-based pipeline developed by the Mackay lab at NC State University, Raleigh, NC (http://dgrp2.gnets.ncsu.edu/) [90,91]. The first step in conducting the GWAS was to adjust line means for the effects of *Wolbachia pipientis* infection as well as the presence of inversions that are segregating in the DGRP. The adjusted line means are then used to fit a linear mixed model, *Y* = **X***b* + **Z***u* + *e*. *Y* is the adjusted phenotypic value, **X** is the design matrix for the fixed variant effect *b*, **Z** is the incidence matrix for the random polygenic effect *u* and *e* is the residual. The vector of polygenic effects *u* has a covariance matrix in the form of **A***σ*^2^, where *σ*^2^ is the polygenic variance component and **A** is the genomic relatedness. Additionally, Manhattan plots were constructed using the qqman package in R [175].

### Functional Assessment of Candidate Genes—Recombination Rate Assay

As described in the text, we selected 20 candidate genes to functionally assess that contained at least one significantly associated genetic variant within them. We selected these genes based on *P*-value of the variant located within or near the gene, effect size of the variant, the number of GWAS that a variant within or near the gene was implicated in and available expression data. To functionally explore these candidate genes with respect to their roles in recombination, we took advantage of available *P*-element insertion lines and chromosomal deletions as well as RNAi lines (S21 Table). Lines containing a *P*-element insertion or chromosomal deletion (deleting the candidate gene) as well as appropriate controls (genetic background used to generate *P*-element insertion or chromosomal deletion) were used in the same crossing scheme (Fig 1) detailed above. For the first cross, ten virgin females from every line containing a *P*-element insertion or chromosomal deletion were crossed to ten doubly-marked males (either *e ro* or *y v*) in eight oz. bottles. Males and females were allowed to mate for five days, after which all adults were cleared from the bottles. For the second cross, ten virgin heterozygous females were collected and backcrossed to ten doubly-marked males in vials. Males and females were allowed to mate for five days, after which all adults were cleared from vials. BC_1_ progeny were collected from each vial, frozen, and scored for sex and for visible phenotypes. For each *P*-element insertion or chromosomal deletion, there were 30 replicates. For each replicate, recombination rates were estimated by taking the ratio of recombinant progeny to the total number of progeny.

The RNAi lines followed an identical crossing scheme except for the males used in the F_0_ cross. These males contained the doubly-marked chromosome (*e ro*) along with *nanos GAL4* driver [176,177]. *nanos* is expressed throughout Drosophila oogenesis [178]. All *P*-element insertions, chromosomal deletions or RNAi lines were compared to appropriate controls using Dunnett’s Test [179,180] using both the raw recombination proportions as well as arcsined transformed data. Statistics were performed in JMP Pro 11.2.0.

### Functional Assessment of Candidate Genes—Expression Analysis

To test the hypothesis that gene expression differences between alleles drive phenotypic variation in recombination rate, we analyzed ovarian mRNA abundance differences between the major and minor allele for each of our 20 candidate genes using quantitative RT-PCR (qPCR). For each candidate gene, three DGRP lines containing the major allele and three DGRP lines containing the minor allele were chosen (S17 Table). For the eight genes that had multiple significant genetic variants associated within the gene region, DGRP lines that contained the most major/minor alleles were selected (S18 Table). For each candidate gene, virgin females were collected from the six DGRP lines contemporaneously to minimize the effects of environmental variation. Females were aged three days in vials with ~0.5 mL of yeast paste. Ovaries were then dissected from anesthetized females in a solution of 1X PBS and stored in Life Technologies RNAlater solution (Life Technologies). For each line, four replicates of ten pairs of ovaries were dissected. Total RNA was extracted from homogenized ovaries using Trizol (Life Technologies) following manufacturer’s instructions. cDNA was generated using Bio-Rad iScript cDNA Synthesis and following manufacturer’s instructions. Primers for candidate genes were generated using FlyPrimerBank [181] (S22 Table). qPCR was run a BioRad CFX384 machine using Bio-Rad iQ SYBR Green following manufacturer's instructions. Four technical replicates for each sample were run on the same 384 plate, minimizing the contribution of between plate variation.

Samples were analyzed using *GAPDH* for normalization due to its relatively consistent expression [182]. For each candidate gene, there were six lines analyzed, three that contained the major allele and three that contained the minor allele identified in our GWAS. For each line, we collected four biological replicates of RNA. We ran four technical replicates of each RNA sample (converted to cDNA). Therefore, for each line, there are a total of 16 qPCR measurements for the candidate gene of interest and 16 qPCR measurements for the *GAPDH* control. Measurements from each DGRP line were normalized by dividing by the average Cq value of *GAPDH* for the corresponding DGRP line, modeled after common normalization procedures [183]. These 96 measurements (48 measurements for the major allele and 48 measurements for the minor allele) were then analyzed by comparing the means of the lines containing the major allele to the means of the lines containing the minor allele via a students t-test using JMP Pro 11.2.0. In addition, the raw Cq values (before normalization) were also analyzed to ensure that potential differential *GAPDH* expression was not biasing results.

## Supporting Information

S1 Text Further discussion of the interchromosomal effect and correlations between recombination rate and other available DGRP phenotypes. (PDF)

S1 Fig Natural variation in recombination rate. Variation in crossover frequency in the all lines of the DGRP in (A) the *e ro* interval on chromosome *3R* and (B) the *y v* interval on the *X* chromosome. The lines for each panel are ordered by recombination rate. Error bars depict standard error. For reference, the reported map distance for the *e ro* interval is 20.4 cM, while the reported map distance for the *y v* interval is 33 cM, denoted by a horizontal line in both graphs.
(TIF)

S2 Fig Scatterplots between replicates and averages. Scatterplots showing pairwise relationships between replicates as well as between each replicate and the overall average for the (A-F) *e ro* and (G-L) *y v* intervals. Spearman’s rho values for all comparisons are included in S3 Table.
(TIF)

S3 Fig Venn diagram of variants uncovered from GWAS. Overlap of significantly associated genetic variants from the three different data sets for each chromosomal interval assayed.
(TIF)

S4 Fig Distribution of effect sizes. Distribution of combined effect sizes for the (A) *e ro* and (B) *y v* intervals from all GWA analyses.
(TIF)

S5 Fig Genome-wide association analyses. Results are depicted for (A) all lines (excluding those with *3R* inversions) for the *e ro* interval and (B) all lines for the *y v* interval. A significance threshold of *P* ≤ 10^−5^ is displayed with a horizontal line. Brackets within the Manhattan plot highlight the chromosomal interval assayed. The triangular heat map displays the amount of linkage disequilibrium (LD, measured here as *r*^2^) between variants. Each major chromosome is depicted. Red denotes complete LD and blue denotes absence of LD.
(TIF)

S6 Fig Genome-wide association analyses. Results are depicted for (A) lines excluding those with *3R* inversions and/or polymorphic inversions for the *e ro* interval and (B) lines excluding those with polymorphic inversions for the *y v* interval. A significance threshold of *P* ≤ 10^−5^ is displayed. Brackets within the Manhattan plot highlight the chromosomal interval assayed. The triangular heat map displays the amount of linkage disequilibrium (LD, measured here as *r*^2^) between variants. Each major chromosome is depicted. Red denotes complete LD and blue denotes absence of LD.
(TIF)

S7 Fig Assessment of expression difference in ovaries via qPCR. For each candidate gene, the raw (before normalization) average expression of three lines with major allele (blue bars) and three lines with minor allele (red bars) are shown. Error bars denote standard error. * indicates a *P* < 0.05, ** indicates *P* < 0.01 and *** indicates *P* < 0.001.
(TIF)

S1 Table Robustness of data. Raw counts, expected numbers and *G*-test *P*-values for number of males versus females, number of wild-type progeny versus doubly marked individuals (*m*_1_ *m*_2_), and number of recombinant progeny (either + *m*_2_ or *m*_1_ +).
(XLSX)

S2 Table Recombination rate data for DGRP lines. Three replicates and average (in cM; used for GWA analysis) for both intervals (*e ro* and *y v*) are included along with inversion status [91].and Wolbachia status of each line [91].
(XLSX)

S3 Table Correlation between replicates. Spearman's rho values for correlations between replicates as well as between each replicate and the overall average for the *e ro* and *y v* intervals.
(XLSX)

S4 Table ANOVA model. ANOVA testing for the effects of Line, Interval, and the interaction of the two. % is the percentage of variance explained by factor.
(XLSX)

S5 Table Correlation between replicates. Spearman's rho values for correlations between replicates as well as between each replicate and the overall average for the *e ro* and *y v* intervals.
(XLSX)

S6 Table Genome-wide association analyses for recombination rate. Results from GWA on *e ro* data set including all DGRP lines (except those containing an inversion on *3R*).
(XLSX)

S7 Table Genome-wide association analyses for recombination rate. Results from GWA on *e ro* data set excluding DGRP lines with an inversion on *3R* or any polymorphic inversions.
(XLSX)

S8 Table Genome-wide association analyses for recombination rate. Results from GWA on *e ro* data set of DGRP lines only with standard karyotypes.
(XLSX)

S9 Table Genome-wide association analyses for recombination rate. Results from GWA on *y v* data set including all DGRP lines.
(XLSX)

S10 Table Genome-wide association analyses for recombination rate. Results from GWA on *y v* data set excluding DGRP lines with any polymorphic inversions.
(XLSX)

S11 Table Genome-wide association analyses for recombination rate. Results from GWA on *y v* data set of DGRP lines only with standard karyotypes.
(XLSX)

S12 Table Description of variants. Breakdown of genetic variants identified in 6 GWAS (see S6 and S11 Tables).
(XLSX)

S13 Table Genetic variants from GWA. List of significantly associated genetic variants within candidate genes with corresponding *P*-value.
(XLSX)

S14 Table Genotypes of DGRP lines. Genotype of each DGRP line at each significantly associated genetic variant within candidate genes surveyed.
(XLSX)

S15 Table Functional assessment results in the *e ro* interval. Raw counts from gene-level assessment in the *e ro* interval.
(XLSX)

S16 Table Functional assessment results in the *y v* interval. Raw counts from gene-level assessment in the *y v* interval.
(XLSX)

S17 Table DGRP lines used for qPCR expression analysis. List of DGRP lines selected for each candidate gene for qPCR expression analysis.
(XLSX)

S18 Table Genotypes of DGRP lines used for qPCR. Genotype of DGRP lines selected for qPCR expression analysis at respective gene of choice.
(XLSX)

S19 Table qPCR expression data. Cq values from qPCR expression analysis, normalized to GAPDH expression. Lines 1–3 for the major and minor allele correspond to lines in S17 Table.
(XLSX)

S20 Table qPCR expression data before normalization. Cq values from qPCR expression analysis. Lines 1–3 for the major and minor allele correspond to lines in S17 Table.
(XLSX)

S21 Table Genotypes of *P*-element insertions, chromosomal deletions and RNAi lines. Lines used for functional assessment.
(XLSX)

S22 Table Primers used for qPCR. Forward and reverse primers of candidate genes for qPCR expression analysis.
(XLSX)

S23 Table Correlation with DGRP phenotypes. Spearman’s rank correlation for crossover rates in the *e ro* and *y v* intervals (using only standard karyotypes) compared to other DGRP published phenotypes. Sources of data are listed both within the main text as well as in the chart. Significant correlations are in bold.
(XLSX)
