# Supplementary material for: The Genetic Architecture of Natural Variation in Recombination Rate in Drosophila melanogaster
Source: PLoS Genet. 2016 Apr 1;12(4):e1005951. doi: 10.1371/journal.pgen.1005951 (PMC4817973; doi:10.1371/journal.pgen.1005951)
Supplement: S1 Text — (PDF) [file pgen.1005951.s001.pdf]

S1 Text. Further discussion of the interchromosomal effect and correlations between recombination rate and other available DGRP phenotypes.

Interchromosomal Effect

Chromosomal inversions were first discovered in *Drosophila melanogaster* [1]. Recombination is suppressed within the inverted region, yet recombination elsewhere in the genome increases through what is known as the interchromosomal effect [2,3]. The interchromosomal effect has been repeatedly documented in Drosophila [4–6], and has also been observed in other species such as grasshopper [7] and maize [8]. A large number of the DGRP lines are either homozygous or polymorphic for a chromosomal inversion, as expected for natural North American populations of *Drosophila melanogaster* [9]. Within the DGRP, there are a total of sixteen different segregating inversions, all on the autosomes [10]. Out of the 205 lines, 93 lines contain at least one inversion.

As described in the text, our recombination rate data from the DGRP are consistent with the interchromosomal effect. Lines with inversions have significantly increased rates of recombination in the *y v* interval relative to lines with standard karyotypes (35.1 cM vs. 31.0 cM, $P < 0.0001$; t-test). This trend is echoed in the *e ro* interval (20.9 cM vs. 20.7 cM) but the difference in recombination frequency between standard and inverted karyotypes is not statistically significant ($P = 0.66$, t-test). These data further confirm the interchromosomal effect in Drosophila.

Correlation with other available DGRP Phenotypes

As a widely-used community resource, the DGRP offers a unique opportunity to examine the relationship between recombination rate and other phenotypes because a variety of phenotypes have been surveyed in this panel. Although anecdotal evidence suggests a link

between recombination rate and organismal fitness [21,22], the adaptive significance of population-level variation in recombination rate remains unknown. We find no direct evidence of a relationship between recombination frequency and fitness in the *e ro* interval but we do see a marginally significant negative correlation in the *y v* interval. The direction of this correlation is opposite of what has been previously reported in humans [22] but similar to what has been reported in Drosophila [21]. It is unclear why humans and Drosophila differ in this way, and whether this is biologically relevant or an artifact of our experimental design. Indeed, our measurement of fitness is coarse and is likely a poor indicator of organismal fitness. Previously reported estimates of longevity in the DGRP, another aspect of organismal fitness, show no significant correlation with our estimates of recombination rate in either interval (**S23 Table**; [16]). Thus, any connection between recombination and fitness based on these data should be interpreted as tenuous at best.

However, if population-level variation in recombination rate has biological significance, one might expect that recombination rate would correlate with other organismal phenotypes. We tested whether crossover rates in the *e ro* or *y v* interval (of lines with standard karyotypes) were correlated with various traits including aggression [11], behavioral response to odorants [12,13], chill coma recovery [14,15], longevity [16], nutritional and immune indices [17], oxidative stress [18], pigmentation [19], sleep phenotypes [20], startle response [14,15], and starvation stress [14,15] (**S23 Table**). The majority of correlations were weak and not statistically significant. However, for the *e ro* interval, crossover rates were significantly positively correlated with female response to citral (Spearman's $\rho = 0.20$, $P = 0.03$). For the *y v* interval, crossover rates were negatively correlated with female and male response to ethyl butyrate (Spearman's $\rho = -0.21$, $P = 0.03$; Spearman's $\rho = -0.20$, $P = 0.04$, respectively) as well as female response to eugenol (Spearman's $\rho = -0.22$, $P = 0.02$). Also in the *y v* interval, similar to the *e ro* interval, crossover rates were positively correlated with female response to citral (Spearman's $\rho = 0.21$, $P = 0.03$), male response to citral (Spearman's $\rho = 0.28$, $P = 0.004$) and also to male response

to hexanal (Spearman's $\rho = 0.20$, $P = 0.04$). While these correlations between crossover rate and behavioral responses to different naturally occurring odorants are statistically significant, the biological link between these phenotypes remains unclear.

The most intriguing significant correlation we uncovered is the correlation of rates of crossing over in the *e ro* interval to female survival time on paraquat-laced food ($\rho = -0.25$, $P = 0.01$). Paraquat can cause oxidative stress and single-base damage, often corrected through the base-excision repair pathway. Though paraquat exposure does not appear to plastically increase meiotic recombination [23], there is clearly a link between stress and recombination in Drosophila and other systems [24–29]. This correlation between recombination and resistance to the toxic effects of oxidative stress specifically in females revealed here may be reflective of the general connection between stress and recombination. Interestingly, of seven candidate genes associated with oxidative stress susceptibility/resistance in the DGRP, two overlap with candidate genes selected for this study, *CG9650* and *Eip75B* [18]. This overlap could suggest conserved players in the DNA damage repair pathway in both meiotically and mitotically dividing cells.

REFERENCES

1. Sturtevant AH. Genetic factors affecting the strength of linkage in Drosophila. Proc Natl Acad Sci U S A. 1917;3: 555–558.

2. Schultz J, Redfield H. Interchromosomal effects on crossing over in Drosophila. Cold Spring Harb Symp Quant Biol. 1951;16: 175–197.

3. Lucchesi JT, Suzuki DT. The Interchromosomal control of recombination. Annu Rev Genet. 1968;2: 53–86.

4. Portin P and MR. Further-Studies on the Interchromosomal Effect on Crossing over in Drosophila melanogaster Affecting the Preconditions of Exchange. Genetica. 1990;82: 203–207.

5. Joyce EF, McKim KS. Chromosome Axis Defects Induce a Checkpoint-Mediated Delay and Interchromosomal Effect on Crossing Over during Drosophila Meiosis. PLoS Genet. 2010;6: e1001059.

6. Stevison LS, Hoehn KB, Noor MAF. Effects of inversions on within- and between-species recombination and divergence. Genome Biol Evol. 2011;3: 830–841.

7. White MJD, Morley FHW. Effects of pericentric rearrangements on recombination in grasshopper chromosomes. Genetics. 1955;40: 604–619.

8. Bellini G, Bianchi A. Interchromosomal effects of inversions on crossover rate in maize. Z Für Vererbungslehre. 1963;94: 126–132. doi:10.1007/BF00895890

9. Mettler LE, Voelker RA, Mukai T. Inversion clines in populations of *Drosophila melanogaster*. Genetics. 1977;87: 169–176.

10. Huang W, Massouras A, Inoue Y, Peiffer J, Rámia M, Tarone A, et al. Natural variation in genome architecture among 205 *Drosophila melanogaster* Genetic Reference Panel lines. Genome Res. 2014;24: 1193–1208. doi:10.1101/gr.171546.113

11. Shorter J, Couch C, Huang W, Carbone MA, Peiffer J, Anholt RRH, et al. Genetic architecture of natural variation in *Drosophila melanogaster* aggressive behavior. Proc Natl Acad Sci. 2015;112: E3555–E3563. doi:10.1073/pnas.1510104112

12. Swarup S, Huang W, Mackay TFC, Anholt RRH. Analysis of natural variation reveals neurogenetic networks for Drosophila olfactory behavior. Proc Natl Acad Sci. 2013;110: 1017–1022. doi:10.1073/pnas.1220168110

13. Arya GH, Magwire MM, Huang W, Serrano-Negron YL, Mackay TFC, Anholt RRH. The genetic basis for variation in olfactory behavior in *Drosophila melanogaster*. Chem Senses. 2015; bjv001. doi:10.1093/chemse/bjv001

14. Ayroles JF, Carbone MA, Stone EA, Jordan KW, Lyman RF, Magwire MM, et al. Systems genetics of complex traits in *Drosophila melanogaster*. Nat Genet. 2009;41: 299–307.

15. Mackay TFC, Richards S, Stone EA, Barbadilla A, Ayroles JF, Zhu D, et al. The *Drosophila melanogaster* Genetic Reference Panel. Nature. 2012;482: 173–178.

16. Ivanov DK, Escott-Price V, Ziehm M, Magwire MM, Mackay TFC, Partridge L, et al. Longevity GWAS using the Drosophila Genetic Reference Panel. J Gerontol A Biol Sci Med Sci. 2015; glv047. doi:10.1093/gerona/glv047

17. Unckless RL, Rottschaefer SM, Lazzaro BP. The complex contributions of genetics and nutrition to immunity in *Drosophila melanogaster*. PLoS Genet. 2015;11: e1005030. doi:10.1371/journal.pgen.1005030

18. Weber AL, Khan GF, Magwire MM, Tabor CL, Mackay TFC, Anholt RRH. Genome-wide association analysis of oxidative stress resistance in *Drosophila melanogaster*. PLoS ONE. 2012;7: e34745. doi:10.1371/journal.pone.0034745

19. Dembeck LM, Huang W, Magwire MM, Lawrence F, Lyman RF, Mackay TFC. Genetic architecture of abdominal pigmentation in *Drosophila melanogaster*. PLoS Genet. 2015;11: e1005163. doi:10.1371/journal.pgen.1005163

20. Harbison ST, McCoy LJ, Mackay TFC. Genome-wide association study of sleep in *Drosophila melanogaster*. BMC Genomics. 2013;14: 1–18.

21. Tucić N, Ayala FJ, Marinković D. Correlation between recombination frequency and fitness in *Drosophila melanogaster*. Genetica. 1981;56: 61–69. doi:10.1007/BF00126931

22. Kong A, Barnard J, Gudbjartsson DF, Thorleifsson G, Jonsdottir G, Sigurdardottir S, et al. Recombination rate and reproductive success in humans. Nat Genet. 2004;36: 1203–1206.

23. Langberg KA. Testing the effects of oxidative stress on genomic recombination in the honey bee, *Apis mellifera* [Internet]. Text, UNCG. 2014. Available: http://libres.uncg.edu/ir/uncg/listing.aspx?styp=ti&id=16578

24. Stern C. An effect of temperature and age on crossing-over in the first chromosome of *Drosophila melanogaster*. Genetics. 1926;12: 530–532.

25. Neel JV. A relation between larval nutrition and the frequency of crossing over in the third chromosome of *Drosophila melanogaster*. Genetics. 1941;26: 506–516.

26. Belyaev DK, Borodin PM. The influence of stress on variation and its role in evolution. Biol Zentralblatt. 1982;100: 705–714.

27. Parsons PA. Evolutionary rates: effects of stress upon recombination. Biol J Linnaan Soc. 1988;35: 49–68.

28. Abdullah MFF, Borts RH. Meiotic recombination frequencies are affected by nutritional states in *Saccharomyces cerevisiae*. PNAS. 2001;98: 14524–14529.

29. Singh ND, Criscoe DR, Skolfield S, Kohl KP, Keebaugh ES, Schlenke TA. Fruit flies diversify their offspring in response to parasite infection. Science. 2015;349: 747–750. doi:10.1126/science.aab1768
