# Supplementary figures and images for: The Genetic Architecture of Natural Variation in Recombination Rate in Drosophila melanogaster
Source: PLoS Genet. 2016 Apr 1;12(4):e1005951. doi: 10.1371/journal.pgen.1005951 (PMC4817973; doi:10.1371/journal.pgen.1005951)

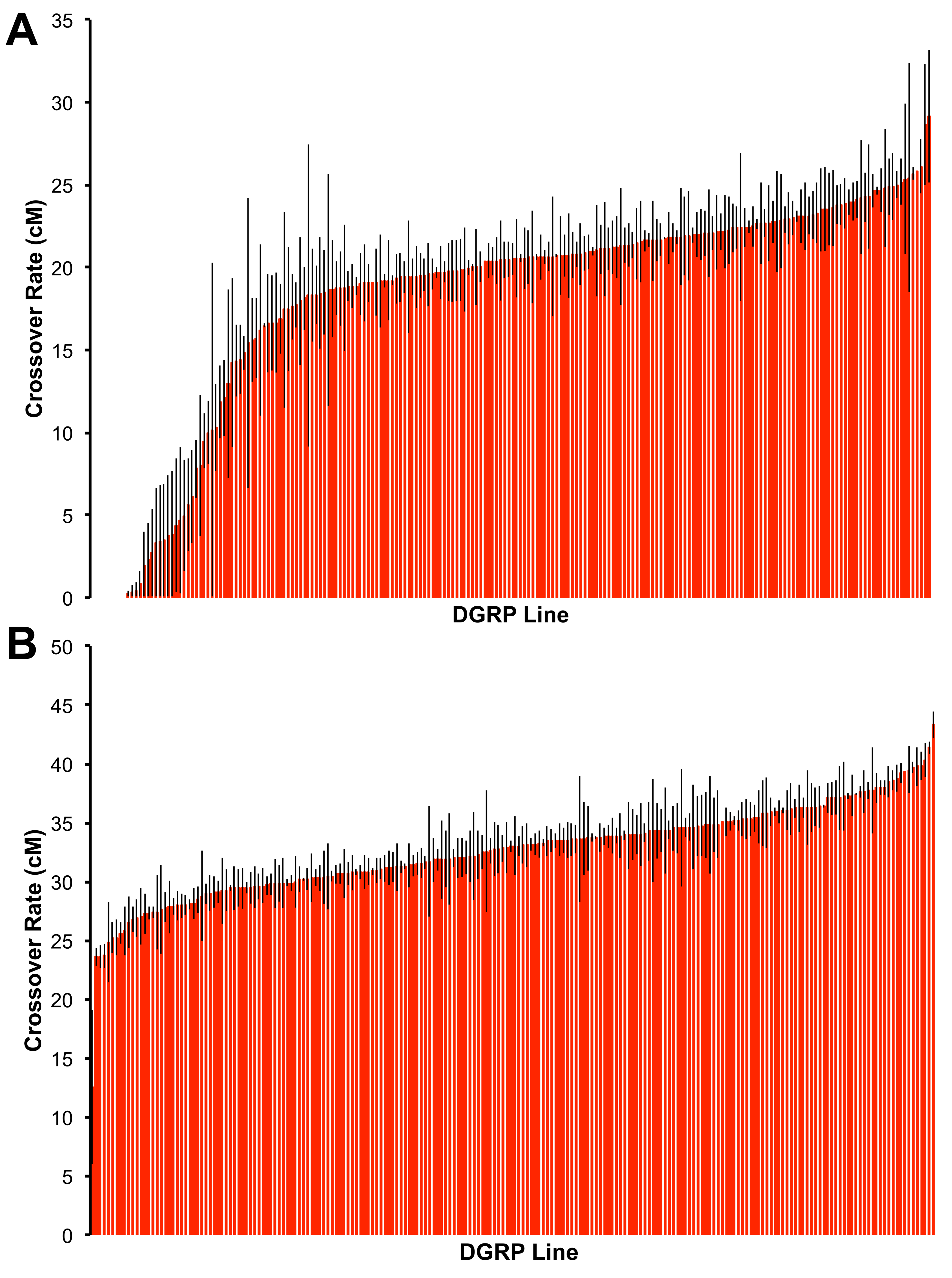

Supplement: S1 Fig — Variation in crossover frequency in the all lines of the DGRP in (A) the e ro interval on chromosome 3R and (B) the y v interval on the X chromosome. The lines for each panel are ordered by recombination rate. Error bars depict standard error. For reference, the reported map distance for the e ro interval is 20.4 cM, while the reported map distance for the y v interval is 33 cM, denoted by a horizontal line in both graphs. (TIF) [file pgen.1005951.s002.tif]

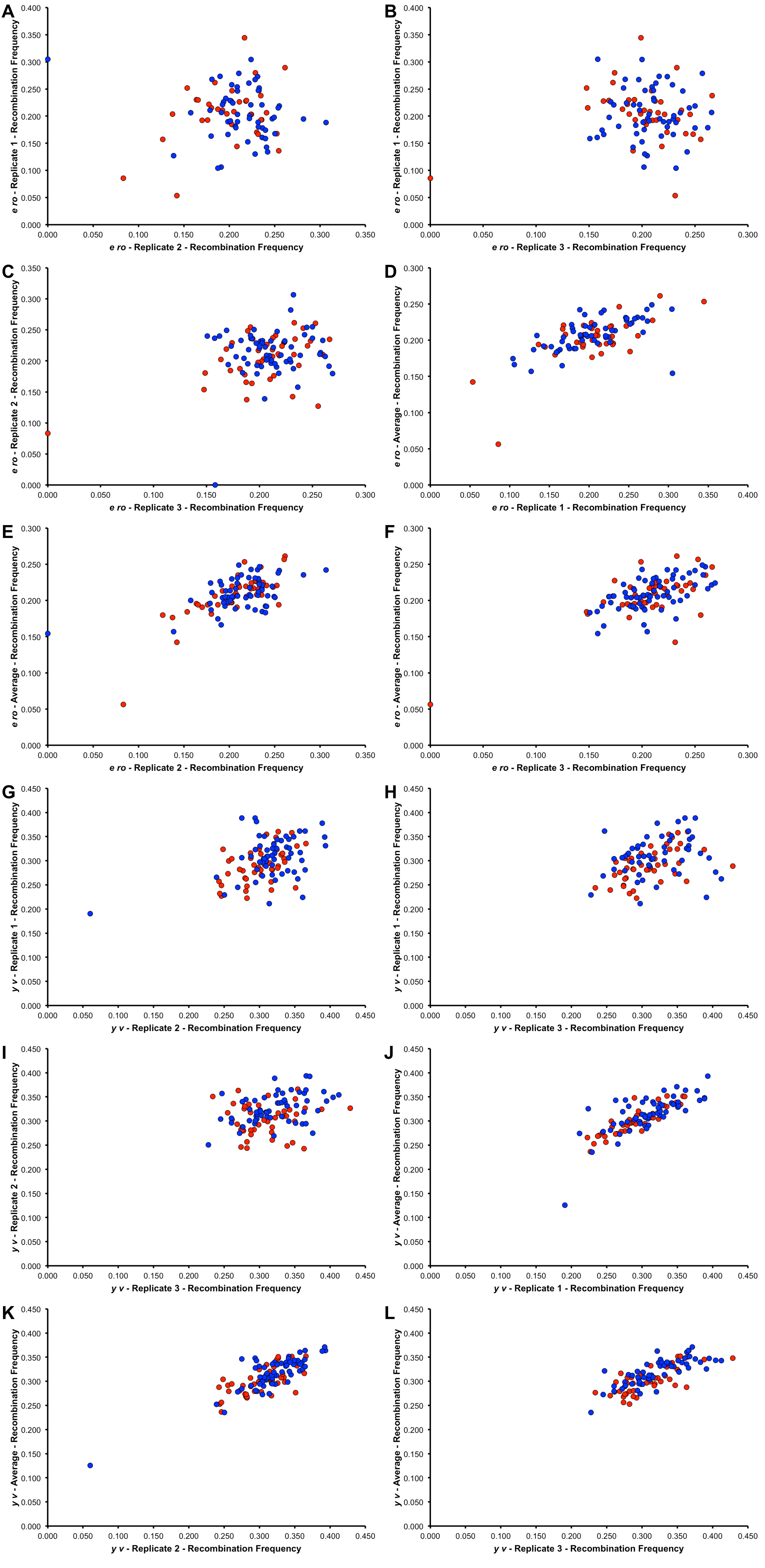

Supplement: S2 Fig — Scatterplots showing pairwise relationships between replicates as well as between each replicate and the overall average for the (A-F) e ro and (G-L) y v intervals. Spearman’s rho values for all comparisons are included in S3 Table. (TIF) [file pgen.1005951.s003.tif]

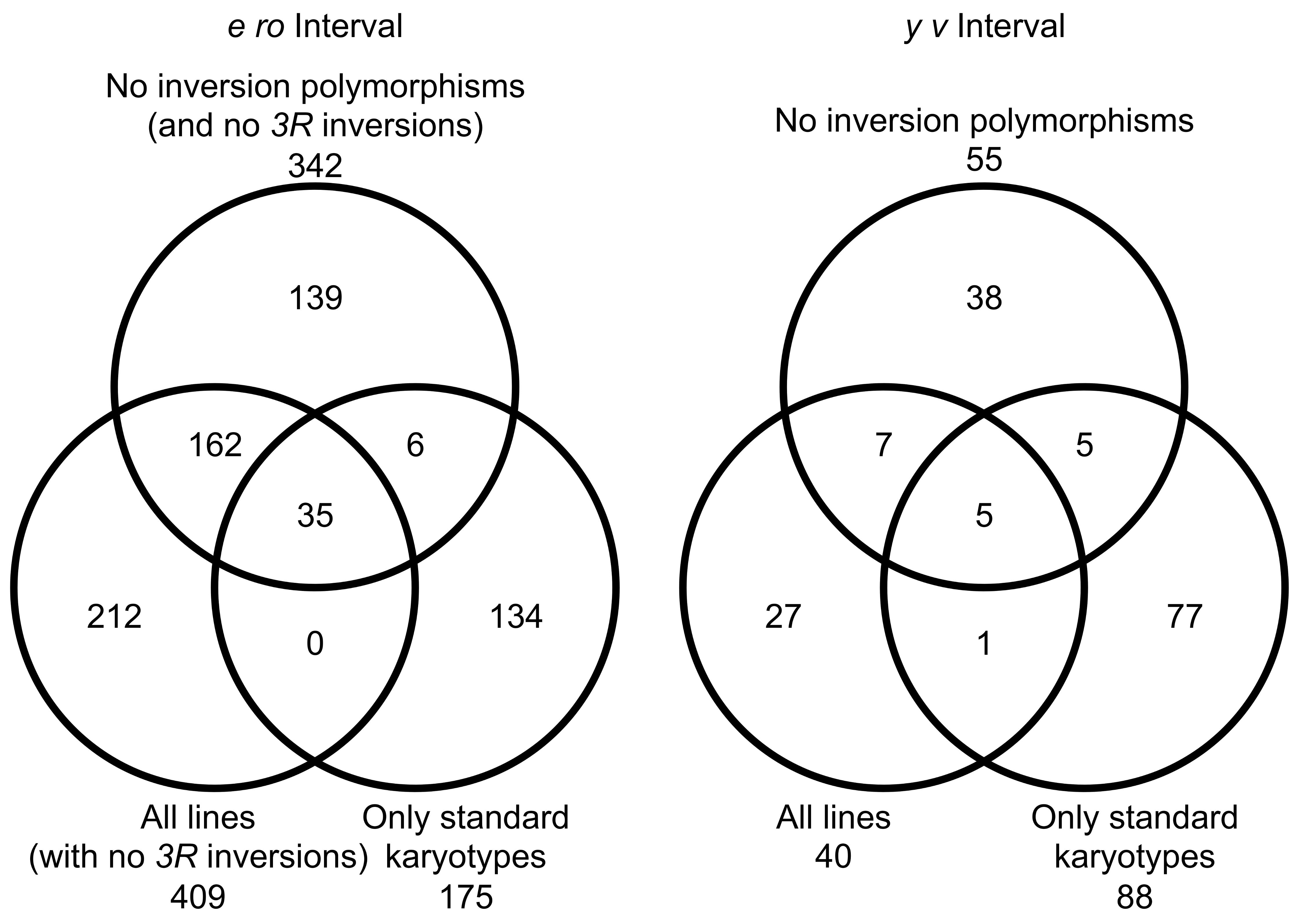

Supplement: S3 Fig — Overlap of significantly associated genetic variants from the three different data sets for each chromosomal interval assayed. (TIF) [file pgen.1005951.s004.tif]

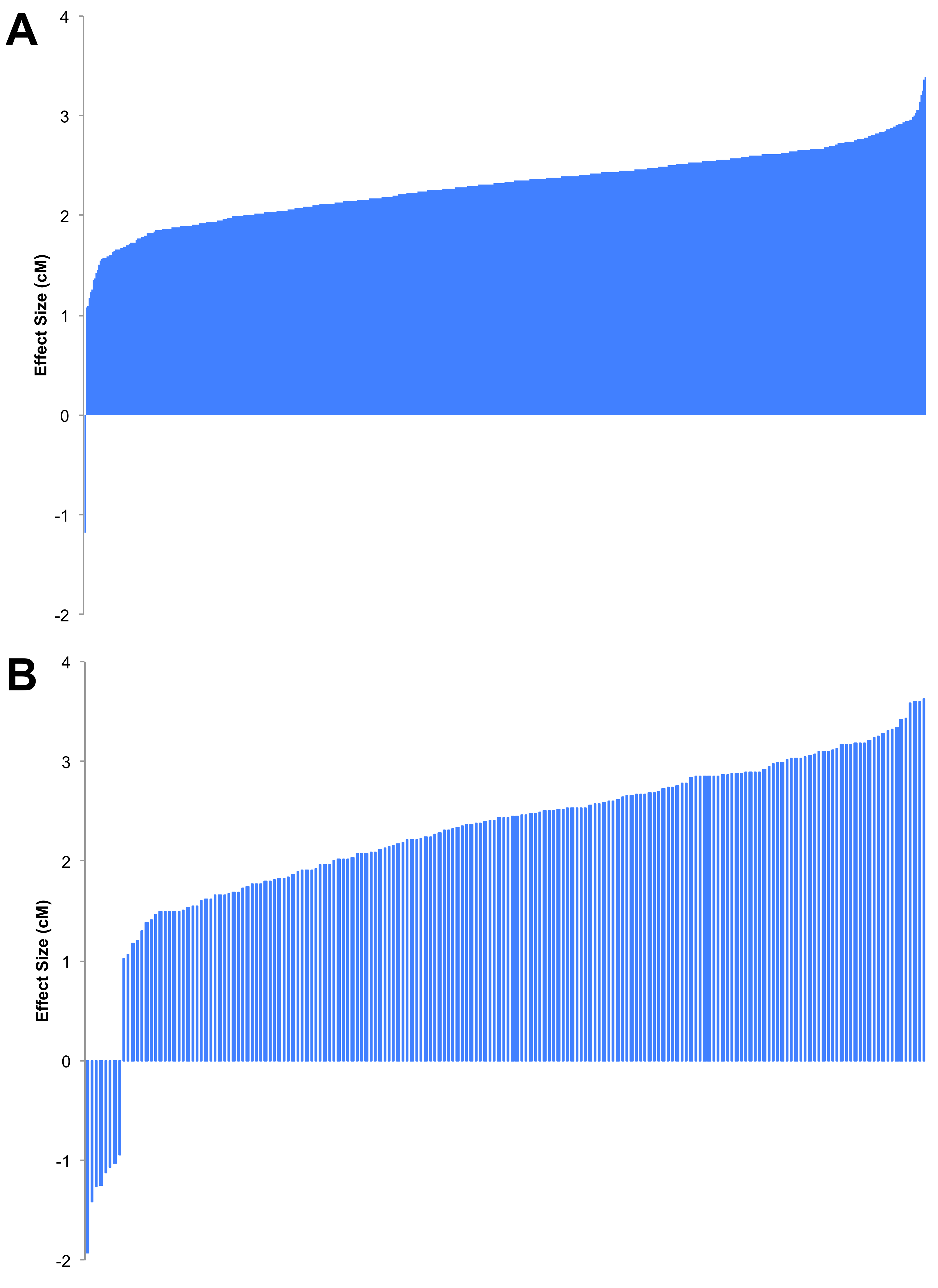

Supplement: S4 Fig — Distribution of combined effect sizes for the (A) e ro and (B) y v intervals from all GWA analyses. (TIF) [file pgen.1005951.s005.tif]

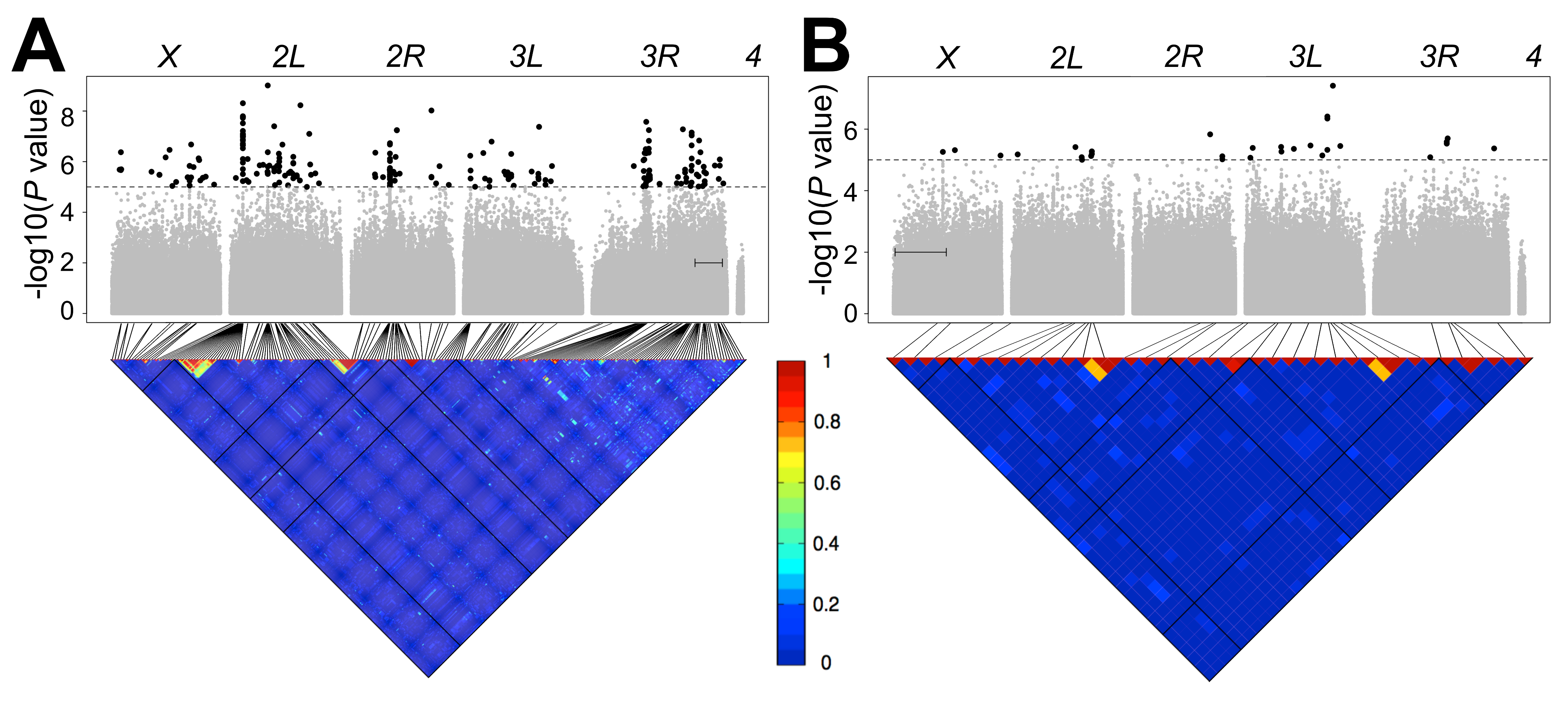

Supplement: S5 Fig — Results are depicted for (A) all lines (excluding those with 3R inversions) for the e ro interval and (B) all lines for the y v interval. A significance threshold of P ≤ 10−5 is displayed with a horizontal line. Brackets within the Manhattan plot highlight the chromosomal interval assayed. The triangular heat map displays the amount of linkage disequilibrium (LD, measured here as r2) between variants. Each major chromosome is depicted. Red denotes complete LD and blue denotes absence of LD. (TIF) [file pgen.1005951.s006.tif]

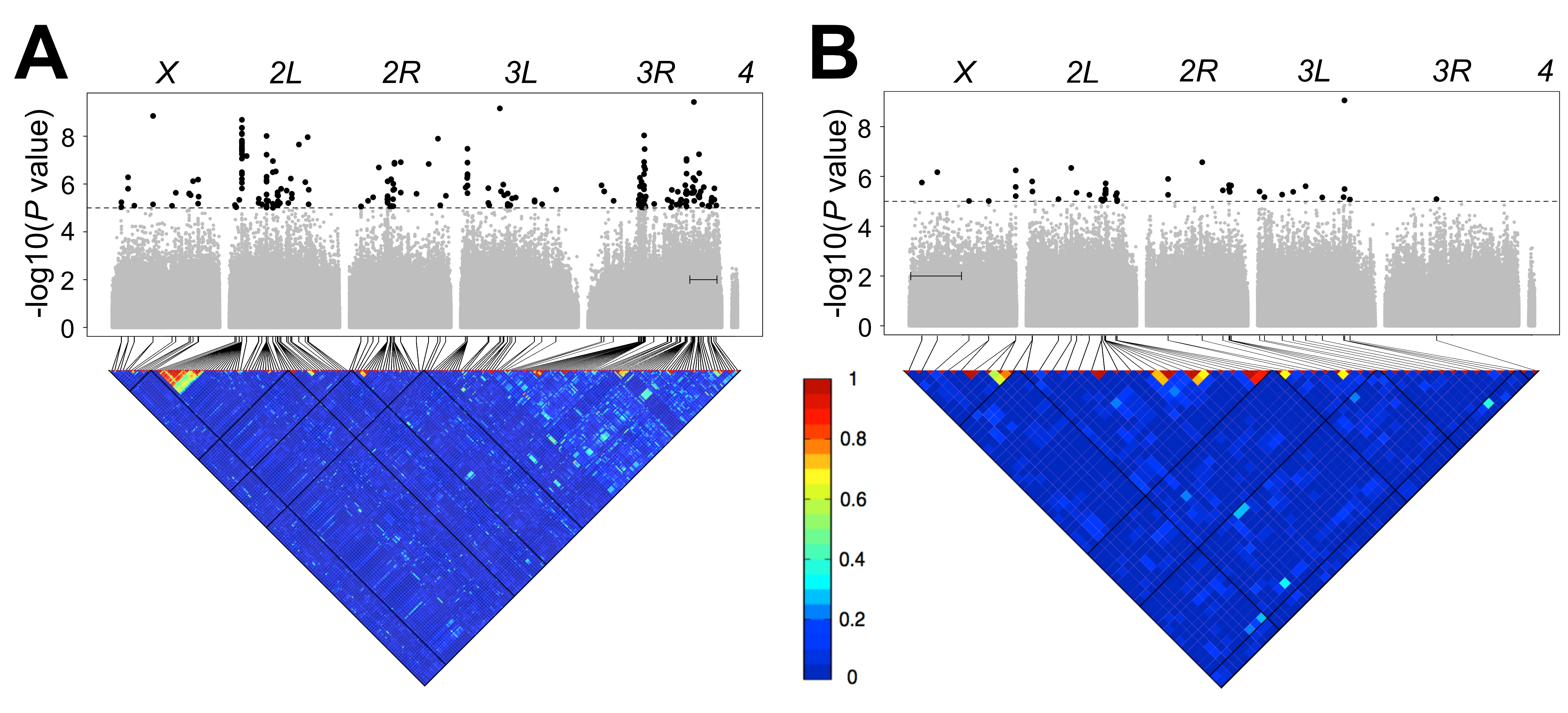

Supplement: S6 Fig — Results are depicted for (A) lines excluding those with 3R inversions and/or polymorphic inversions for the e ro interval and (B) lines excluding those with polymorphic inversions for the y v interval. A significance threshold of P ≤ 10−5 is displayed. Brackets within the Manhattan plot highlight the chromosomal interval assayed. The triangular heat map displays the amount of linkage disequilibrium (LD, measured here as r2) between variants. Each major chromosome is depicted. Red denotes complete LD and blue denotes absence of LD. (TIF) [file pgen.1005951.s007.tif]

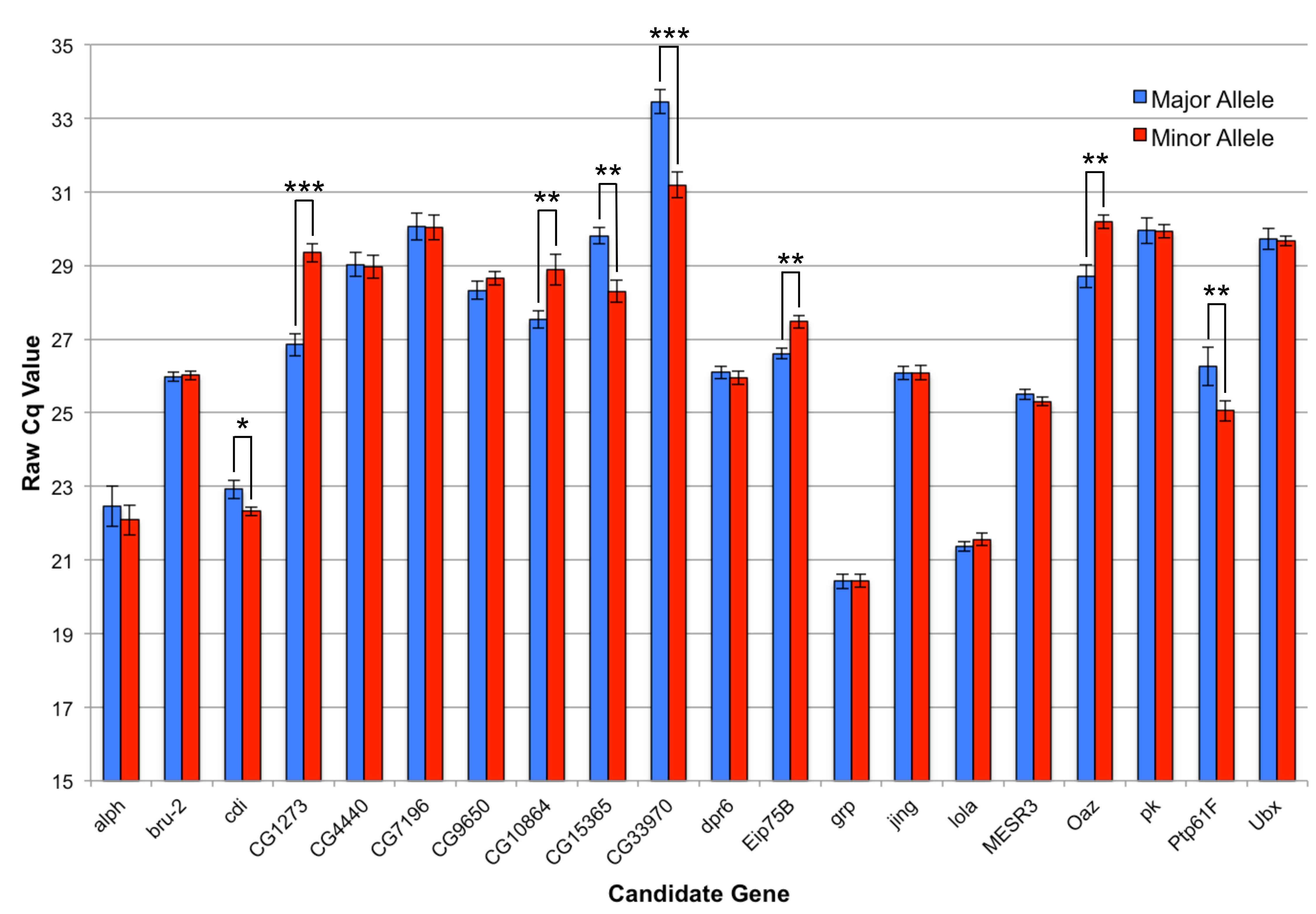

Supplement: S7 Fig — For each candidate gene, the raw (before normalization) average expression of three lines with major allele (blue bars) and three lines with minor allele (red bars) are shown. Error bars denote standard error. * indicates a P < 0.05, ** indicates P < 0.01 and *** indicates P < 0.001. (TIF) [file pgen.1005951.s008.tif]
